# Values and preferences of patients with inborn errors of immunity and their caregivers: a systematic review

**DOI:** 10.3389/fimmu.2026.1876316

**Published:** 2026-07-22

**Authors:** Daniel G. Rayner, Michael Nakhla, Leonardo Ologundudu, Veronika Sustrova, Jenny Garkaby, Rongbo Zhu

**Affiliations:** 1 Schulich School of Medicine and Dentistry, Western University, London, ON, Canada; 2 Michael G. DeGroote School of Medicine, McMaster University, Hamilton, ON, Canada; 3 David R. Cheriton School of Computer Science, University of Waterloo, Waterloo, ON, Canada; 4 Division of Clinical Immunology, Allergy and Dermatology, Department of Pediatrics, McMaster University, Hamilton, ON, Canada; 5 Division of Clinical Immunology and Allergy, Department of Medicine, Western University, London, ON, Canada

**Keywords:** inborn errors of immunity, patient preferences, primary immunodeficiencies, systematic review, thematic synthesis

## Abstract

Understanding patient values and preferences can provide valuable insight and help improve patient care for patients with inborn errors of immunity (IEIs). Therefore, this review sought to systematically review the values and preferences of patients with IEIs and their caregivers towards the management of IEIs. We searched MEDLINE, Embase, PsycINFO, and CINAHL from database inception up to June 7, 2026, for studies addressing the values and preferences of patients with IEIs or their caregivers. Paired reviewers independently screened citations, extracted data, and assessed risk of bias. We used iterative thematic content analysis to qualitatively synthesize our findings and the GRADE-CERQual approach to rate the confidence of the evidence supporting each theme. Fifty-two studies enrolled 6,388 participants. We identified 6 key themes: (1) Patients are probably more willing to engage with and accept treatment if counseled by healthcare professionals knowledgeable in IEIs (moderate confidence). Patients probably place a high value on IEI treatments that (2) minimize needle pricks and adverse effects, and (3) maximize independence, efficiency, and flexibility (moderate confidence). (4) Patients probably place a high value on effective transitions from pediatric to adult care services (moderate confidence). (5) Patients may have significant concerns about the financial impact of IEI management (low confidence). (6) Patients may place a high value on the integration of mental health and social support into their care (low confidence). Ultimately, these 6 key themes on the values and preferences of patients with IEIs and their caregivers may inform optimal clinical practice and future research in IEIs.

**Systematic review registration:**

https://www.crd.york.ac.uk/PROSPERO/, identifier CRD420251114032.

## Introduction

Inborn errors of immunity (IEIs), formerly referred to as primary immunodeficiencies (PIDs), are a large heterogenous class of over 550 genetic disorders characterized by reduced or absent function in one or more components of the immune system (1, 2). Over 6 million adults and children globally (3) are estimated to be affected by IEIs, impacting approximately 1 in 1200 live births (4). Patients with IEIs often experience chronic or recurrent infectious and non-infectious manifestations, and often require life-long treatment, which can include immunoglobulin replacement therapy (subcutaneous or intravenous), antimicrobial prophylaxis, hematopoietic stem cell transplantation, gene therapy, and other targeted immunomodulators (5, 6). Clinicians often leverage clinical practice guidelines to engage patients during the shared decision-making process, however, existing guidelines for the management of IEIs (5, 7–10) have only informally considered patient and caregiver values and preferences, without explicitly incorporating patient and caregiver perspectives towards IEI care and the aforementioned treatments.

Understanding patient and caregiver values and preferences in IEI management can improve the rigor and applicability of clinical practice guidelines and future research. Thus, we conducted a systematic review to summarize and synthesize the values and preferences of patients with IEIs and their caregivers towards the management of IEIs.

## Methods

We conducted this review following similar methods to previous systematic reviews of patient values and preferences (11–16), as described below, and we report our findings in accordance with the Preferred Reporting Items for Systematic Reviews and Meta-analyses (PRISMA) statement (17) (see **Supplementary Material 1** for the completed PRISMA reporting checklist). We prospectively registered our review protocol on PROSPERO (CRD420251114032).

### Search strategy and selection criteria

We systematically searched MEDLINE, Embase, CINAHL, and PsycINFO from database inception to June 7, 2026, for primary research studies of any design (qualitative, quantitative, or mixed-methods) addressing the values and preferences of patients with IEIs or their caregivers (see **Supplementary Material 2** for the complete search strategies used and the inception dates reported by each database). To identify further potentially eligible studies, we additionally searched the reference lists of the included studies. We included studies reporting on the values and preferences of patients or caregivers regarding IEI management. We did not impose any restrictions based on language of publication or publication status.

### Study selection, data extraction, and risk of bias assessment

Paired reviewers (DGR, MN, LO, VS) screened titles and abstracts and reviewed full-text articles independently using Covidence (Veritas Health Innovation, Melbourne, Australia). Thereafter, paired reviewers (DGR, MN, LO, VS) extracted data independently using standardized and pre-piloted Excel extraction forms. Reviewers resolved disagreements by consensus, and if necessary, through consultation with a third reviewer. We extracted bibliographic information (author names, year of publication, publication status, country), study design, patient and/or caregiver characteristics (sample size, age, gender), values and preferences data (relevant quotes, reported estimates, and measures of variability), and sources of funding. When studies presented quantitative data only in figures, we extracted data using WebPlotDigitizer v4.6 (https://automeris.io/WebPlotDigitizer).

Paired reviewers (DGR, MN, LO, VS) assessed the risk of bias and quality of each included study, using the Critical Appraisal Skills Programme (CASP) checklist (18) for studies reporting qualitative data, and a risk of bias tool (19) developed by the Grading of Recommendations Assessment, Development and Evaluation (GRADE) working group (20) for studies reporting quantitative data.

### Data synthesis

We leveraged iterative thematic synthesis to synthesize the extracted data from the included studies (21, 22). To facilitate the merging of quantitative and qualitative values and preferences data, we used a critical meta-narrative synthesis (23) to transform quantitative data into qualitative data through systematic profiles and asking critical questions (see **Supplementary Material 3** for detailed examples of these transformations). Through an iterative process, two reviewers (DGR, MN) independently reviewed the extracted qualitative data and transformed quantitative data to generate codes, consolidated similar codes to derive descriptive themes, and combined similar descriptive themes to develop overall analytical themes. At each step of the thematic synthesis process, the reviewers held research meetings to triangulate their findings, and considered the extent to which each theme was supported by patient- or caregiver-reported data. To promote readers’ understanding of the type of data contributing to each theme, we present representative quotes for each developed overall analytical theme.

### Rating the confidence in the evidence

For each derived overall analytical theme, we rated the confidence in the evidence from the qualitative evidence syntheses using the GRADE-CERQual (Grading of Recommendations Assessment, Development and Evaluation-Confidence in the Evidence from Reviews of Qualitative Research) approach (24). Using the GRADE-CERQual approach, the confidence in the evidence from a qualitative evidence synthesis starts as high, and may be rated down if there are concerns with respect to the methodological limitations (risk of bias), coherence (consistency across studies), adequacy (quantity and richness of the data), or relevance (indirectness of the study populations) of the studies included in the thematic synthesis.

## Results

**Figure 1:**
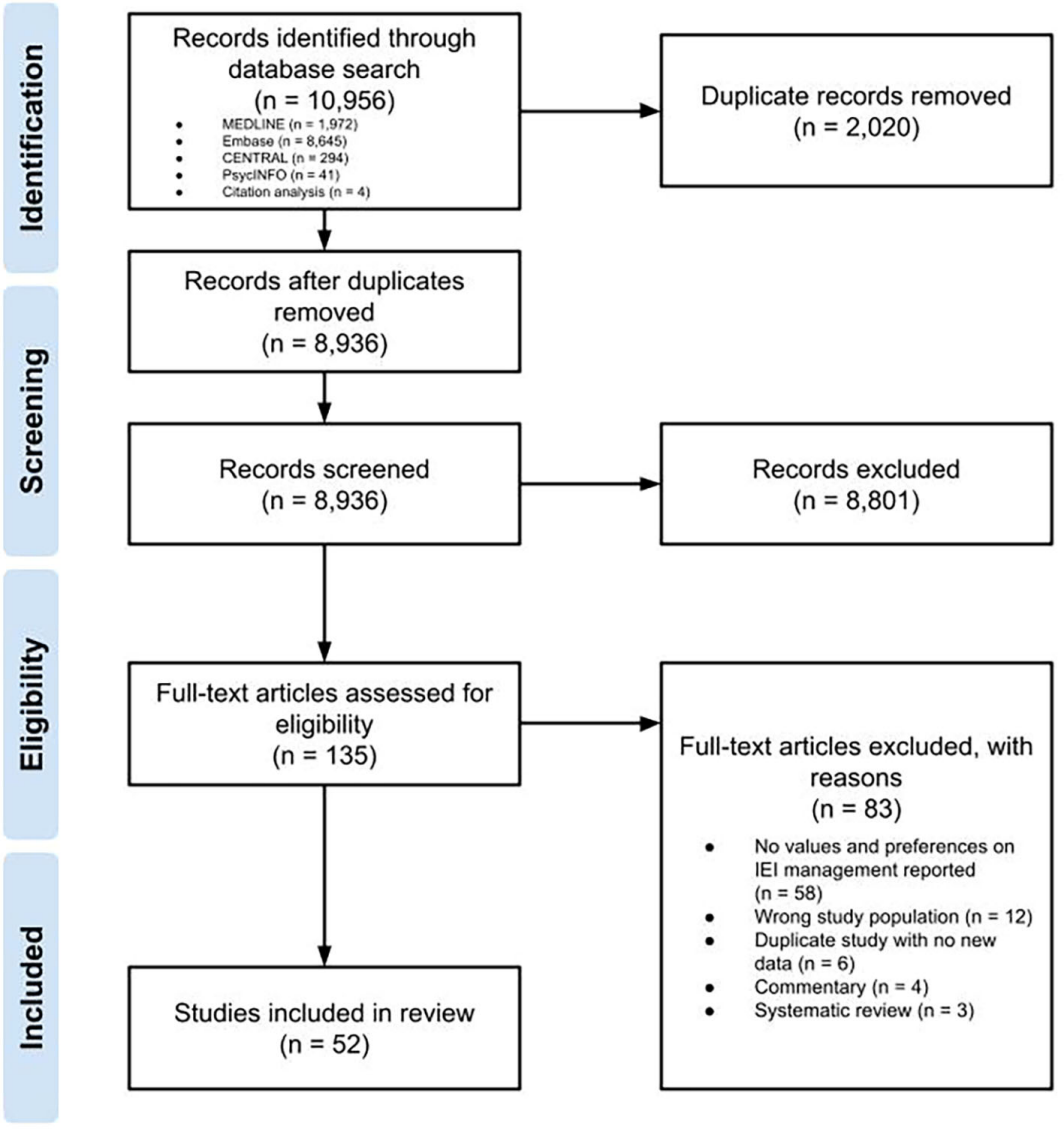
PRISMA study selection and flow diagram for systematic review of patient and caregiver values and preferences for the management of inborn errors of immunity.

Through our systematic search, we screened 8,936 unique titles and abstracts, reviewed 135 potentially eligible full-text articles, and ultimately included 52 studies (25–76) reporting on the values and preferences of 6,388 participants (**Figure 1**).

**Table 1** presents the characteristics of the included studies. Sixteen (31%) studies were qualitative, 28 were (54%) quantitative, and 8 (15%) leveraged a mixed-methods design. Nearly two-thirds of studies (n=34, 65%) elicited the perspectives of patients only, 8 (15%) elicited the perspectives of caregivers only, and 10 (19%) elicited both the perspectives of patients and caregivers. Three studies (6%) enrolling 165 participants did not report the exact breakdown of patients and caregivers among their participants; the remaining 49 studies enrolled 5,638 patients and 585 caregivers. These studies included a median of 51 patients (range: 3–1317) and 18 caregivers (range: 3–145). The included studies had a median of mean patient age of 32.3 years (range: 0.4–58.0) and a median of 59% (range: 12–100%) patients were women. Fourteen studies (27%) reported funding from industry sources.

**Table 1:** Characteristics of included studies (n=52).

Study	Country^±^	Design	N Participants	Participant breakdown	Condition	Patient characteristics		Funding source
						Age (years) mean (SD), range	Women (%)	
Aberumand 2022 (25)	Canada	Survey	449	449 Patients	Any IEI	NR, 18+	75%	Non-Industry
Agnihotri 2021 (26)	USA	Survey	35	17 Patients 18 Caregivers	Any IEI	NR	NR	None
AhmadAzahari 2023 (27)	Malaysia	Focus Groups	15	15 Caregivers	NR	NR	NR	Non-Industry
AhmedMeelad 2022 (28)	Malaysia	Interviews	10	10 Caregivers	NR	16.7 (10.2), 6–41	NR	Non-Industry
Allen 2022 (29)	USA	Survey	15	15 Patients	Any IEI	NR, 13–41	NR	Non-Industry
Beers 2025 (30)	USA	Interviews	20	20 Patients	Any IEI	32.3 (6.5), 18+	65%	Non-Industry
Chatelier 2014 (31)	Australia	Survey	61	61 Patients	Any IEI	NR	NR	NR
Clark 2025 (32)	Australia	Interviews	17	17 Caregivers	Any IEI	NR	NR	Non-Industry
Cozon 2018 (33)	France	Interviews & Focus Groups	8	8 Patients	Any IEI	58.0 (22.0), 31–80	88%	Industry
Davidson 2024 (34)	USA	Interviews	23	23 Patients	GATA2 deficiency, PIK3CD gain of-function disorder, or CTLA4 deficiency	32.0 (NR), 18–48	61%	Non-Industry
Duff 2014 (35)	USA	Survey	131	131 Patients	Humoral IEI	NR	NR	NR
Eltan 2022 (36)	Turkiye	Survey	21	21 Patients	Any IEI	8.8 (4.4), NR	48%	None
Espanol 2012 (37)	NR	Survey	300	216 Patients 84 Caregivers	Any IEI	NR	NR	NR
Espanol 2014 (38)	Multiple	Survey	300	216 Patients 84 Caregivers	Any IEI	NR	59%	Industry
Franzese 2025 (39)	USA	Remote Ethnography	11	11 Patients	Any IEI	NR, 18+	91%	Industry
Gardulf 1995 (40)	Multiple	Survey	152	152 Patients	PAD	NR, 18–76	59%	Non-Industry
Gardulf 2004 (41)	Multiple	Survey	47	47 Patients	PAD	NR, 3–74	26%	Non-Industry
Geoffroy 2025 (42)	USA	Survey	42	42 Patients	Any IEI	NR	79%	NR
Gonzalez 2022 (43)	USA	DCE	119	119 Patients	PAD	48.5 (14.0), 18–77	87%	Industry
Hansen 2004 (44)	Sweden	Survey	9	9 Patients	PAD	NR, 25–43	100%	NR
Ito 2014 (45)	USA	Survey	51	51 Patients	Any IEI	NR, 16+	NR	Industry
Janssen 2021 (46)	the Netherlands	Interviews	14	14 Patients	PAD	45.0 (15.2), 16–68	79%	None
Johansen 2026 (47)	Denmark	Interviews	9	9 Patients	Any IEI	NR	NR	Industry
Jones 2020a (48)	Multiple	Survey	395	395 Patients	Any IEI	45.9 (14.6), 18–83	63%	Industry
Jones 2020b (49)	UK	Interviews	30	30 Patients	Any IEI	52.9 (17.5), 27–92	57%	Industry
Joseph 2016 (50)	USA	Focus Groups	5	5 Caregivers	NR	NR	NR	Non-Industry
Karali 2025 (51)	Turkiye	Survey	169	169 Patients	Any IEI	NR, 1–79	45%	None
Keestra 1988 (52)	the Netherlands	Survey	87	87 Patients	Any IEI	20.3 (NR), 1–70	29%	Non-Industry
Kutsa 2022 (53)	USA	Interviews	26	26 Caregivers	NR	0.4 (NR), NR	NR	Non-Industry
Lechanska-Helman 2020 (54)	Poland	Survey	23	23 Caregivers	NR	3.9 (1.2), 1–5	52%	Non-Industry
Machado 2024 (55)	Multiple	Interviews & Survey	14	Breakdown of Patients & Caregivers NR	Primary HLH	NR	NR	Industry
Margolis 2017 (56)	USA	Interviews	33	33 Patients	CGD	23.0 (NR), 19–27	12%	Non-Industry
Meckley 2020 (57)	Multiple	Survey	49	49 Patients	Any IEI	NR, 2–67	39%	Industry
Mohamed 2012 (58)	USA	DCE	318	252 Patients 66 Caregivers	Any IEI	50.2 (13.8), 19–80	75%	Industry
Myers 2025 (59)	USA	Survey	300	300 Patients	Any IEI	53.0 (NR), NR	81%	NR
NapiorkowskaBaran 2021 (60)	Poland	Survey	85	85 Patients	Any IEI	40.2 (13.9), 18+	43%	Non-Industry
Ouimet 2023 (61)	Canada	Interviews	15	9 Patients 6 Caregivers	Any IEI	21.4 (NR), NR	NR	Non-Industry
Pergent 2023 (62)	Multiple	Survey	1317	1317 Patients	Any IEI	47.0 (16.6), 12–100	74%	Non-Industry
Ponsford 2015 (63)	UK	Survey	14	14 Patients	CVID, Hypogammaglobulinemia, SPAD, Good’s syndrome	43.4 (15.2), 21–67	43%	NR
Pursey 2025 (64)	Australia	Interviews	7	7 Caregivers	NR	NR	NR	Non-Industry
Raspa 2020 (65)	USA	Interviews & Survey	76	76 Caregivers	NR	10.9 (10.6), NR	NR	Non-Industry
Reid 2014 (66)	Canada	Survey	91	Breakdown of Patients & Caregivers NR	Any IEI	23.0 (15.0), NR	38%	Non-Industry
Runken 2016 (67)	USA	Survey	152	152 Patients	Any IEI	NR	NR	NR
Runken 2017 (68)	USA	Survey	158	158 Patients	Any IEI	NR	NR	NR
Rutland 2025 (69)	USA	Survey	9	9 Patients	Any IEI	43.1 (13.3), 19–61	56%	None
Schwaneck 2025 (70)	Germany	Survey	70	70 Patients	Any IEI	NR, 19–71	57%	Industry
Smith 2021 (71)	USA	Survey	151	6 Patients 145 Caregivers	SCID	NR	30%	Non-Industry
Sowers 2022 (72)	USA	Survey	511	511 Patients	Any IEI	NR, 18+	94%	None
Suleymanova 2026 (73)	Russia	Survey	200	200 Patients	Any IEI	NR, 18+	63%	None
Sultan 2017 (74)	Canada	Survey	60	Breakdown of Patients & Caregivers NR	Any IEI	11.7 (5.7), 0–18	40%	Industry
Wasserman 2017 (75)	USA	Survey	158	158 Patients	Any IEI	NR	NR	NR
Wolf 2026 (76)	Multiple	Interviews	6	3 Patients 3 Caregivers	APDS	NR	NR	Industry

APDS, Activated phosphoinositide 3-kinase delta syndrome; CGD, Chronic granulomatous disease; CVID, Common variable immunodeficiency; DCE, Discrete choice experiment; HLH, Hemophagocytic lymphohistiocytosis; IEI, Inborn error of immunity; NR, Not reported; PAD, Predominantly antibody deficiency; SCID, Severe combined immunodeficiency; SPAD, Specific antibody deficiency; UK, United Kingdom; USA, United States of America.

± As self-described by the authors.

Most included studies had limitations regarding their applied methodology. Among the qualitative studies, the most common source of risk of bias was the lack of reporting of sufficiently rigorous qualitative data analyses. Among the quantitative studies, the most common limitation was researchers failing to ensure that patients fully understood the instrument or survey being used (see **Supplementary Materials 4**-**5** for the full risk of bias assessments).

### Patient and caregiver values and preferences

We identified 6 key themes related to the values and preferences of patients with IEIs and their caregivers towards the management of IEIs. In the section below, we present each theme alongside representative quotes (see **Supplementary Materials 6**-**11** for the full qualitative thematic synthesis for each theme, including all leveraged quotes). We observed consistent findings between patients with IEIs and their caregivers. **Table 2** presents the GRADE-CERQual summary of findings for each theme. **Supplementary Material 12** presents the GRADE-CERQual ratings for each domain and their justifications.

**Table 2:** GRADE-CERQual summary of findings of key themes.

Review finding	Number of studies (number of participants)	Confidence in evidence	Explanation of GRADE–CERQual assessment^±^	Associated references
Patients and caregivers are probably more willing to engage with and accept treatment if counseled by healthcare professionals knowledgeable in IEIs	24 (1020)	Moderate	Moderate concerns with methodological limitations. Minor concerns with coherence.	(26–30, 32, 34, 39, 40, 44, 46, 49, 50, 52, 53, 55, 56, 61, 64–66, 71, 75, 76)
Patients and caregivers probably place a high value on IEI treatments that minimize needle pricks and adverse effects	14 (1920)	Moderate	Moderate concerns with methodological limitations.	(36–40, 43, 48, 49, 53, 54, 58, 63, 71, 74)
Patients and caregivers probably place a high value on IEI treatments that maximize independence, efficiency, and flexibility	26 (2730)	Moderate	Moderate concerns with methodological limitations. Minor concerns with coherence.	(28, 31, 33, 36–41, 43, 45, 47–49, 54, 56–60, 63, 65, 66, 68, 69, 74)
Patients and caregivers probably place a high value on effective transitions from pediatric to adult care services	24 (1020)	Moderate	Moderate concerns with methodological limitations.	(26–30, 32, 34, 39, 40, 44, 46, 49, 50, 52, 53, 55, 56, 61, 64–66, 71, 75, 76)
Patients and caregivers may have significant concerns about the financial impact of IEI management	8 (262)	Low	Moderate concerns with methodological limitations and adequacy. Minor concerns with coherence.	(27, 28, 32, 34–36, 49, 61)
Patients and caregivers may place a high value on the integration of mental health and social supports into their care	7 (203)	Low	Moderate concerns with methodological limitations and adequacy.	(30, 34, 46, 49, 56, 64, 65)

GRADE-CERQual = Grading of Recommendations Assessment, Development and Evaluation-Confidence in the Evidence from Reviews of Qualitative Research; IEI = Inborn error of immunity.

± The GRADE-CERQual approach involves assessment of 4 components: (1) Methodological limitations (the extent to which there are concerns about the design or conduct of the primary studies that contribute evidence); (2) Coherence (how clear and cogent the fit is between the data from the primary studies and a review finding that synthesizes that data); (3) Adequacy (an overall determination of the degree of richness and quantity of data supporting a review finding); and (4) Relevance (the extent to which the body of evidence from the primary studies supporting a review finding is applicable to the context [perspective or population, phenomenon of interest, setting] specified in the review question).

### Theme 1: counseling from clinicians knowledgeable in IEIs

Thematic synthesis of 24 studies (1,020 participants) (26–30, 32, 34, 39, 40, 44, 46, 49, 50, 52, 53, 55, 56, 61, 64–66, 71, 75, 76) found that patients and caregivers are probably more willing to engage with and accept treatment if counseled by healthcare professionals who are knowledgeable in IEIs (moderate confidence). Below are illustrative quotes from three of the included studies showcasing the importance that patients and their caregivers place on counseling from knowledgeable IEI clinicians:

“If you know more about your illness, you can live your life better. … If you don’t know much about your illness, you keep struggling and keep asking, ‘Why is this happening [ … ] to me? What is happening? Why do I get all those infections?’” (Patient) (30)

“We just had to have faith in his doctors and ask a lot of questions. They were the ones that always calmed us down and told us, like, ‘this is our plan. He’s going to be okay. We really think that this is going to work.’ We really just had to listen to them.” (Caregiver) (53)

### Theme 2: minimizing treatment adverse effects and needle pricks

Thematic synthesis of 14 studies (1,920 participants) (36–40, 43, 48, 49, 53, 54, 58, 63, 71, 74) found that patients and caregivers probably place a high value on IEI treatments that minimize needle pricks and adverse effects (moderate confidence). Patients and caregivers often accepted and prioritized treatments, most often various immunoglobulin replacement therapy approaches, that limited the number of needle pricks and adverse effects and harms (e.g., swelling, scarring, pruritus):

“Independently of the method, to prick myself with the needle is the worst thing.” (Patient) (40)

“I get everything prepared and I do it, and it does help, visualizing it in that way. I hate afterwards when I’ve finished, the site area is really sore, it’s swollen and because I’ve got a scar from my belly button down.” (Patient) (49)

### Theme 3: maximizing treatment independence, efficiency, and flexibility

Thematic synthesis of 26 studies (2,730 participants) (28, 31, 33, 36–41, 43, 45, 47–49, 54, 56–60, 63, 65, 66, 68, 69, 74) found that patients and caregivers probably place a high value on IEI treatments that maximize independence, efficiency, and flexibility (moderate confidence). Patients and caregivers often felt burdened by treatment regimens that required travel to hospitals or additional storage procedures, valuing simple treatment regimens that can be self-administered and conveniently incorporated into their weekly schedules:

“It’s took a lot less time, not having to go to hospital. You haven’t got to waste a day, or waste half a day in the hospital, wasting doctor and nurses[‘] time … but now I just go to work, come home one day, have my tea, and then start this treatment straight afterwards.” (Patient) (49)

“Home treatment gives you the opportunity to live like a ‘normal’ human being. If anyone feels disabled by the antibody deficiency, home treatment reduces this feeling to a minimum.” (Patient) (40)

“It is more bothersome when I have to travel, planning material, storing the product in a cool place.” (Patient) (33)

### Theme 4: effective transitions from pediatric to adult care services

Thematic synthesis of 24 studies (1,020 participants) (26–30, 32, 34, 39, 40, 44, 46, 49, 50, 52, 53, 55, 56, 61, 64–66, 71, 75, 76) found that patients and caregivers probably place a high value on effective transitions from pediatric to adult care services (moderate confidence). Patients and caregivers reflected on their personal care transition experiences and emphasized the preference for clinicians to counsel and engage with patients and caregivers on the transition process at younger ages to ensure effective and smooth transitions in care:

“Start young, like around 15 or 16, a few years before. Talk about their flights and appointments with their parents there but not doing it for them.” (Patient) (56)

“Encourage patients to be an active participant. It’s important to know what and why.” (Patient) (56)

“I’m just telling you that we would have felt … Less nervous. Because when they tell us: ‘You’re going to meet [adult immunologist].’ But who is he? Are we going to feel comfortable? Will we like him? Will we feel understood? We’ve been in the void for eighteen years.” (Caregiver) (61)

### Theme 5: concerns about the financial impact of IEIs

Thematic synthesis of 8 studies (262 participants) (27, 28, 32, 34–36, 49, 61) found that patients and caregivers may have significant concerns about the financial impact of IEI management (low confidence). Patients often described the financial burden of living with IEIs, including direct (e.g., treatment costs) and indirect costs (e.g., leave of absence from work, travel costs):

“I had another job three years ago and they just didn’t understand. I asked for a leave of absence for the hospital, and they fired me because it was too much for them.” (Patient) (61)

“I’ve had opportunities to go over to America, working over there, and I’m like I’ve got to have treatment, and travel insurance and that, they’re not going to cover the cost of this treatment. For me to pay for it, it’s probably really, from a financial standpoint- it’s just causing me a lot of problems really.” (Patient) (49)

“When I was young, I used to say, ‘I can’t wait to be 18 and an adult’, but what a shitty idea I had. [ … ] You know, when you’re an adult, you have bills to pay, you work to pay your bills, to pay for food and then rent. I’m working right now to pay for medicine, my rent, my food. What do I have left? 25 dollars a week. It’s a shame. [ … ] That’s basic survival. Right now, that’s the only thing I’m doing, surviving.” (Patient) (61)

### Theme 6: integrating mental health and social support into care

Thematic synthesis of 7 studies (203 participants) (30, 34, 46, 49, 56, 64, 65) found that patients and caregivers may place a high value on the integration of mental health and social support into their care (low confidence). Patients and caregivers described the mental toll that IEIs had on their lives and their subsequent desire for support from mental health professionals and those with similar lived experiences:

“I think it’s best to have similar people your age ease you into the situation. Talking to someone personal would be really helpful. Someone to connect with on your level that knows what you’re going through. I’ve found it useful to talk to other patients with CGD [chronic granulomatous disease].” (Patient) (56)

“Diving into therapy and learning how to manage stress and all the self-care aspects for sure so I wouldn’t spiral and get sick. … We need to figure out what I need to do to help myself.” (Patient) (30)

## Discussion

This systematic review and qualitative thematic synthesis of 52 studies, including 6,388 participants, found 6 key themes related to the values and preferences of patients and caregivers that may guide optimal IEI management. Moderate confidence evidence showed that patients and their caregivers probably place a high value on counseling from knowledgeable IEI clinicians, and counseling from these clinicians probably increases their willingness to engage with and accept treatment. Moderate confidence evidence also found that patients and caregivers probably preferred treatment regimens that minimized needle pricks and adverse effects, while also promoting patient independence, efficiency, and flexibility. Additionally, patients and their caregivers probably placed a high importance on smooth and effective care transitions from pediatric to adult services, preferring that clinicians counseled and engaged with patients and caregivers on the transition process at younger ages. Low confidence evidence suggested that patients and their caregivers may have a significant concern about the direct and indirect costs of IEI management. Finally, low confidence evidence also suggested that patients and caregivers may highly value formal and informal support resources, including peer support groups and professional mental health services, to be incorporated into their IEI care. The primary reasons for rating down the confidence in the evidence for most themes were methodological limitations (risk of bias) and adequacy (quantity and richness) of the data.

### Strengths, relation to previous work, and implications for clinical practice

To our knowledge, this is the first systematic review and thematic synthesis to summarize the values and preferences of patients and their caregivers towards the management of IEIs. Key strengths of our review include (1): leveraging a comprehensive search strategy to identify all relevant published and unpublished studies on patient and caregiver values and preferences, (2) using standardized approaches to merge qualitative and quantitative data, improving the precision and robustness of our analyses, and (3) conducting all qualitative syntheses independently and in duplicate with triangulation at each step to minimize the impact of personal bias on the findings and to promote reproducibility.

The findings of our systematic review align with previous research on the values and preferences of patients and caregivers in other chronic conditions, including asthma (77), aortic stenosis (12), acute episodic migraine (78), and chronic pain (79). Across chronic conditions, patients consistently place a high value on avoiding treatment adverse effects and harms, as well as counseling and guidance on potential treatment options from trusted clinicians who are knowledgeable on the condition. Our findings are also consistent with previous research comparing subcutaneous and intravenous therapies in rheumatoid arthritis (80) and oncological conditions (81), with patients frequently preferring subcutaneous over intravenous administration due to the greater flexibility and productivity that self-administration with subcutaneous therapy offers to patients and their caregivers.

Currently, potential treatment regimens for patients with IEIs include immunoglobulin replacement therapy (subcutaneous or intravenous), antimicrobial prophylaxis, hematopoietic stem cell transplantation, gene therapy, and targeted therapies such as abatacept and other immunomodulators (5). Informed by this review, clinicians should extensively counsel patients and their caregivers on the potential benefits and harms of each treatment, including potential adverse effects and logistical burdens, its expected financial impact, and its congruency with patient autonomy; this may be done through individualized discussions or by leveraging shared decision-making tools for IEI care (82). Furthermore, the transition of care from pediatric to adult care services continues to be a challenge for patients with IEIs, with barriers including fragmented adult care and limited access to adult specialists (83). Given the importance that patients and caregivers place on effective care transitions, there is a need for greater adoption of formal, standardized guidelines on the care transition process for patients with IEIs, such as the European Reference Network on Rare Immunodeficiency, Autoinflammatory and Autoimmune Disease (ERN-RITA) (84) or the Italian Primary Immunodeficiency Network (7) care transition guidelines.

### Limitations and implications for research

Our systematic review has limitations. Firstly, many of the included qualitative studies had important limitations, including insufficient reporting of whether the relationship between the researcher and the participant was considered, and of whether the qualitative data analyses were sufficiently rigorous. We addressed these limitations by considering them during our standardized GRADE-CERQual ratings, assessing the confidence in the evidence for each theme. Second, most studies focused on the values and preferences of patients and their caregivers towards immunoglobulin replacement therapy, with studies seldom assessing their perspectives towards other treatments, including antimicrobial prophylaxis or hematopoietic stem cell transplantation. Future research should investigate patient and caregiver values and preferences towards these treatments. Third, the included studies seldom evaluated the perspectives of patients with specific IEIs, with most studies broadly recruiting patients with IEIs (n=33, 63%) or predominantly antibody deficiencies (n=5, 10%); as such, we were unable to analyze themes based on International Union of Immunological Societies (IUIS) classification. Although we found consistent findings across the included studies, regardless of whether they broadly recruited patients with IEIs or recruited patients with specific IEIs, it should be noted that IEIs are a very heterogenous group of conditions, and our findings may not apply equally to all IEI subtypes. Fourth, our systematic search may have not fully captured all available grey literature and unpublished studies on patient values and preferences in IEI management. Fifth, while males are more frequently affected by inborn errors of immunity, women were more prevalent among the participants of the included studies in our review, which may not fully represent the patient population in clinical practice. This may reflect known survey response patterns, whereby females, older individuals, those with higher educational attainment, individuals who are married or cohabiting, and those with personal experience of the condition under study are more likely to participate in medical questionnaires (85). Lastly, most studies identified were conducted in North America, Western Europe, or Australia, reflecting the perspectives of patients and their caregivers from these countries, and our findings may not be generalizable to all possible cultures or settings, including low- and middle-income countries. Future research should ensure that the perspectives of individuals from low- to middle-income countries are represented.

## Conclusion

In this first systematic review, to our knowledge, summarizing patient and caregiver values and preferences towards the management of IEIs, we identified 6 key themes that can be used to inform optimal shared decision-making, future research, and clinical practice guidelines. In the management of IEIs, patients and their caregivers value counseling from clinicians who are knowledgeable in IEIs, treatments that minimize needle pricks and adverse effects, treatments that maximize independence, efficiency, and flexibility, effective transitions from pediatric to adult care, and the integration of mental health and social support into their care. Patients and their caregivers also have significant concerns about the financial impact of IEI management.

## Data Availability

The original contributions presented in the study are included in the article/**Supplementary Material**. Further inquiries can be directed to the corresponding author.
