# Supplementary material for: Values and preferences of patients with inborn errors of immunity and their caregivers: a systematic review
Source: Front Immunol. 2026 Jul 22;17:1876316. doi: 10.3389/fimmu.2026.1876316 (PMC13437357; doi:10.3389/fimmu.2026.1876316)
Supplement: Supplementary file 1 [file Table1.docx]

**Supplementary Material 1.** Completed PRISMA 2020 checklist.

| **Section and Topic** | **Item #** | **Checklist item** | **Location where item is reported** |
| --- | --- | --- | --- |
| **TITLE** | | |  |
| Title | 1 | Identify the report as a systematic review. | Title |
| **ABSTRACT** | | |  |
| Abstract | 2 | See the PRISMA 2020 for Abstracts checklist. | Abstract Text |
| **INTRODUCTION** | | |  |
| Rationale | 3 | Describe the rationale for the review in the context of existing knowledge. | Introduction Text |
| Objectives | 4 | Provide an explicit statement of the objective(s) or question(s) the review addresses. | Introduction Text |
| **METHODS** | | |  |
| Eligibility criteria | 5 | Specify the inclusion and exclusion criteria for the review and how studies were grouped for the syntheses. | Methods Text |
| Information sources | 6 | Specify all databases, registers, websites, organizations, reference lists and other sources searched or consulted to identify studies. Specify the date when each source was last searched or consulted. | Methods Text |
| Search strategy | 7 | Present the full search strategies for all databases, registers and websites, including any filters and limits used. | Supplementary Material 2 |
| Selection process | 8 | Specify the methods used to decide whether a study met the inclusion criteria of the review, including how many reviewers screened each record and each report retrieved, whether they worked independently, and if applicable, details of automation tools used in the process. | Methods Text |
| Data collection process | 9 | Specify the methods used to collect data from reports, including how many reviewers collected data from each report, whether they worked independently, any processes for obtaining or confirming data from study investigators, and if applicable, details of automation tools used in the process. | Methods Text |
| Data items | 10a | List and define all outcomes for which data were sought. Specify whether all results that were compatible with each outcome domain in each study were sought (e.g. for all measures, time points, analyses), and if not, the methods used to decide which results to collect. | Methods Text |
|  | 10b | List and define all other variables for which data were sought (e.g. participant and intervention characteristics, funding sources). Describe any assumptions made about any missing or unclear information. | Methods Text |
| Study risk of bias assessment | 11 | Specify the methods used to assess risk of bias in the included studies, including details of the tool(s) used, how many reviewers assessed each study and whether they worked independently, and if applicable, details of automation tools used in the process. | Methods Text |
| Effect measures | 12 | Specify for each outcome the effect measure(s) (e.g. risk ratio, mean difference) used in the synthesis or presentation of results. | Methods Text |
| Synthesis methods | 13a | Describe the processes used to decide which studies were eligible for each synthesis (e.g. tabulating the study intervention characteristics and comparing against the planned groups for each synthesis (item #5)). | Methods Text |
|  | 13b | Describe any methods required to prepare the data for presentation or synthesis, such as handling of missing summary statistics, or data conversions. | Methods Text, Supplementary Material 3 |
|  | 13c | Describe any methods used to tabulate or visually display results of individual studies and syntheses. | Methods Text |
|  | 13d | Describe any methods used to synthesize results and provide a rationale for the choice(s). If meta-analysis was performed, describe the model(s), method(s) to identify the presence and extent of statistical heterogeneity, and software package(s) used. | Methods Text |
|  | 13e | Describe any methods used to explore possible causes of heterogeneity among study results (e.g. subgroup analysis, meta-regression). | Methods Text |
|  | 13f | Describe any sensitivity analyses conducted to assess robustness of the synthesized results. | Methods Text |
| Reporting bias assessment | 14 | Describe any methods used to assess risk of bias due to missing results in a synthesis (arising from reporting biases). | Not Applicable |
| Certainty assessment | 15 | Describe any methods used to assess certainty (or confidence) in the body of evidence for an outcome. | Methods Text |
| Study selection | 16a | Describe the results of the search and selection process, from the number of records identified in the search to the number of studies included in the review, ideally using a flow diagram. | Results Text, Figure 1 |
|  | 16b | Cite studies that might appear to meet the inclusion criteria, but which were excluded, and explain why they were excluded. | Figure 1 |
| Study characteristics | 17 | Cite each included study and present its characteristics. | Results Text, Table 1 |

| **Section and Topic** | **Item #** | **Checklist item** | **Location where item is reported** |
| --- | --- | --- | --- |
| **RESULTS** | |  |  |
| Risk of bias in studies | 18 | Present assessments of risk of bias for each included study. | Supplementary Material 4-5 |
| Results of individual studies | 19 | For all outcomes, present, for each study: (a) summary statistics for each group (where appropriate) and (b) an effect estimate and its precision (e.g. confidence/credible interval), ideally using structured tables or plots. | Table 2 |
| Results of syntheses | 20a | For each synthesis, briefly summarize the characteristics and risk of bias among contributing studies. | Results Text, Supplementary Material 12 |
|  | 20b | Present results of all statistical syntheses conducted. If meta-analysis was done, present for each the summary estimate and its precision (e.g.  confidence/credible interval) and measures of statistical heterogeneity. If comparing groups, describe the direction of the effect. | Table 2, Supplementary Material 6-11 |
|  | 20c | Present results of all investigations of possible causes of heterogeneity among study results. | Results Text |
|  | 20d | Present results of all sensitivity analyses conducted to assess the robustness of the synthesized results. | Results Text |
| Reporting biases | 21 | Present assessments of risk of bias due to missing results (arising from reporting biases) for each synthesis assessed. | Not Applicable |
| Certainty of evidence | 22 | Present assessments of certainty (or confidence) in the body of evidence for each outcome assessed. | Table 2, Supplementary Material 12 |
| **DISCUSSION** | |  |  |
| Discussion | 23a | Provide a general interpretation of the results in the context of other evidence. | Discussion Text |
|  | 23b | Discuss any limitations of the evidence included in the review. | Discussion Text |
|  | 23c | Discuss any limitations of the review processes used. | Discussion Text |
|  | 23d | Discuss implications of the results for practice, policy, and future research. | Discussion Text |
| **OTHER INFORMATION** | |  |  |
| Registration and protocol | 24a | Provide registration information for the review, including register name and registration number, or state that the review was not registered. | Methods Text |
|  | 24b | Indicate where the review protocol can be accessed, or state that a protocol was not prepared. | Methods Text |
|  | 24c | Describe and explain any amendments to information provided at registration or in the protocol. | Methods Text |
| Support | 25 | Describe sources of financial or non-financial support for the review, and the role of the funders or sponsors in the review. | See funding statement |
| Competing  interests | 26 | Declare any competing interests of review authors. | See disclosures statement |
| Availability of data, code and other materials | 27 | Report which of the following are publicly available and where they can be found: template data collection forms; data extracted from included studies; data used for all analyses; analytic code; any other materials used in the review. | See data sharing statement |

**Supplementary Material 2.** Search strategies – from database inception to June 7^th^, 2026

| **MEDLINE** | | |
| --- | --- | --- |
| **Database(s): Ovid MEDLINE(R) ALL <1946 to June 05, 2026>** | | |
| **Search Strategy:** | | **Number of Records** |
| 1 | exp Attitude to Health/ | 516078 |
| 2 | exp Patient Participation/ | 32159 |
| 3 | exp Patient Preference/ | 13055 |
| 4 | preference*.ti,ab. | 236931 |
| 5 | (choice or choices).ti. | 51178 |
| 6 | value*.ti. | 223511 |
| 7 | health state values.ti,ab. | 222 |
| 8 | valuation*.ti. | 2248 |
| 9 | expectation*.ti,ab. | 133982 |
| 10 | attitude*.ti,ab. | 231159 |
| 11 | acceptab*.ti,ab. | 285084 |
| 12 | knowledge.ti,ab. | 1120567 |
| 13 | point of view.ti,ab. | 53544 |
| 14 | user participation.ti,ab. | 419 |
| 15 | users participation.ti,ab. | 85 |
| 16 | patient participation.ti,ab. | 3478 |
| 17 | patients participation.ti,ab. | 871 |
| 18 | patient perspective*.ti,ab. | 8352 |
| 19 | patients perspective*.ti,ab. | 10088 |
| 20 | user perspective*.ti,ab. | 1044 |
| 21 | users perspective*.ti,ab. | 985 |
| 22 | patient perce*.ti,ab. | 8594 |
| 23 | patients perce*.ti,ab. | 14652 |
| 24 | health perception*.ti,ab. | 3945 |
| 25 | user perce*.ti,ab. | 987 |
| 26 | users perce*.ti,ab. | 1631 |
| 27 | user view*.ti,ab. | 156 |
| 28 | users view*.ti,ab. | 554 |
| 29 | patient?? view*.ti,ab. | 4462 |
| 30 | ((decision* and mak*).ti. or decision mak*.ti,ab. or decisions mak*.ti,ab.) and (patient* or user* or men or women).ti,ab. | 161290 |
| 31 | user?? perspective*.ti,ab. | 1975 |
| 32 | user?? perce*.ti,ab. | 2519 |
| 33 | discrete choice*.ti,ab. | 4670 |
| 34 | decision board*.ti,ab. | 47 |
| 35 | decision analy*.ti,ab. | 10587 |
| 36 | decision-support.ti,ab. | 30338 |
| 37 | decision tool*.ti,ab. | 1664 |
| 38 | decision aid*.ti,ab. | 5432 |
| 39 | discrete-choice*.ti,ab. | 4670 |
| 40 | exp Decision Making/ and (patient* or user* or men or women).ti. | 26082 |
| 41 | exp Decision Support Systems, Clinical/ or exp Decision Support Techniques/ | 97806 |
| 42 | (health and utilit*).ti. | 2391 |
| 43 | gamble*.ti,ab. | 5832 |
| 44 | prospect theory.ti,ab. | 524 |
| 45 | preference score*.ti,ab. | 739 |
| 46 | preference elicitation.ti,ab. | 365 |
| 47 | health utilit*.ti,ab. | 3336 |
| 48 | utility value*.ti,ab. | 2994 |
| 49 | utility score*.ti,ab. | 2417 |
| 50 | utility estimate*.ti,ab. | 396 |
| 51 | health state.ti,ab. | 6227 |
| 52 | feeling thermometer*.ti,ab. | 95 |
| 53 | best-worst scaling.ti,ab. | 513 |
| 54 | time trade-off.ti,ab. | 1590 |
| 55 | TTO.ti,ab. | 1842 |
| 56 | probability trade-off.ti,ab. | 21 |
| 57 | preference based.ti,ab. | 2088 |
| 58 | multiattribute.ti,ab. | 463 |
| 59 | multi attribute.ti,ab. | 1214 |
| 60 | EuroQol 5D.ti,ab. | 2169 |
| 61 | EuroQol5D.ti,ab. | 29 |
| 62 | EQ5D.ti,ab. | 1144 |
| 63 | EQ 5D.ti,ab. | 17146 |
| 64 | SF6D.ti,ab. | 49 |
| 65 | SF 6D.ti,ab. | 1031 |
| 66 | HUI.ti,ab. | 1974 |
| 67 | 15D.ti,ab. | 2170 |
| 68 | (patient* adj (value* or satisfact*)).ti,ab. | 69443 |
| 69 | or/1-68 | 2814775 |
| 70 | exp Primary Immunodeficiency Diseases/ | 1696 |
| 71 | (primary adj3 (immunodeficienc* or deficienc*)).mp. | 13177 |
| 72 | exp Common Variable Immunodeficiency/ | 2808 |
| 73 | (common variable immunodeficiency or CVID).mp. | 4414 |
| 74 | exp Granulomatous Disease, Chronic/ | 3508 |
| 75 | (chronic granulomatous disease or CGD).mp. | 4869 |
| 76 | exp Severe Combined Immunodeficiency/ | 4758 |
| 77 | severe combined immunodeficienc*.mp. | 8804 |
| 78 | exp Lymphohistiocytosis, Hemophagocytic/ | 4747 |
| 79 | hemophagocytic lymphohistiocytosis.mp. | 5607 |
| 80 | ((IVIG or intravenous immunoglobulin) adj3 replace*).mp. | 333 |
| 81 | (antibod* adj3 deficienc*).mp. | 2580 |
| 82 | hyper IgM syndrome.mp. | 583 |
| 83 | (inborn error* adj3 immunity).mp. | 2484 |
| 84 | or/70-83 | 38967 |
| 85 | 69 and 84 | 1972 |
| **Embase** | | |
| **Database(s): Embase Classic+Embase <1974 to 2026 June 04>** | | |
| **Search Strategy:** | | **Number of Records** |
| 1 | exp Attitude to Health/ | 149861 |
| 2 | exp Patient Participation/ | 43980 |
| 3 | exp Patient Preference/ | 35874 |
| 4 | preference*.ti,ab. | 305534 |
| 5 | (choice or choices).ti. | 65268 |
| 6 | value*.ti. | 302466 |
| 7 | health state values.ti,ab. | 292 |
| 8 | valuation*.ti. | 3211 |
| 9 | expectation*.ti,ab. | 173125 |
| 10 | attitude*.ti,ab. | 299703 |
| 11 | acceptab*.ti,ab. | 412606 |
| 12 | knowledge.ti,ab. | 1447133 |
| 13 | point of view.ti,ab. | 87581 |
| 14 | user participation.ti,ab. | 489 |
| 15 | users participation.ti,ab. | 86 |
| 16 | patient participation.ti,ab. | 5390 |
| 17 | patients participation.ti,ab. | 1738 |
| 18 | patient perspective*.ti,ab. | 13454 |
| 19 | patients perspective*.ti,ab. | 15468 |
| 20 | user perspective*.ti,ab. | 1238 |
| 21 | users perspective*.ti,ab. | 1132 |
| 22 | patient perce*.ti,ab. | 14151 |
| 23 | patients perce*.ti,ab. | 22703 |
| 24 | health perception*.ti,ab. | 5590 |
| 25 | user perce*.ti,ab. | 1071 |
| 26 | users perce*.ti,ab. | 1745 |
| 27 | user view*.ti,ab. | 253 |
| 28 | users view*.ti,ab. | 689 |
| 29 | patient?? view*.ti,ab. | 6791 |
| 30 | ((decision* and mak*).ti. or decision mak*.ti,ab. or decisions mak*.ti,ab.) and (patient* or user* or men or women).ti,ab. | 251821 |
| 31 | user?? perspective*.ti,ab. | 2318 |
| 32 | user?? perce*.ti,ab. | 2726 |
| 33 | discrete choice*.ti,ab. | 6634 |
| 34 | decision board*.ti,ab. | 65 |
| 35 | decision analy*.ti,ab. | 14943 |
| 36 | decision-support.ti,ab. | 40978 |
| 37 | decision tool*.ti,ab. | 2669 |
| 38 | decision aid*.ti,ab. | 8200 |
| 39 | discrete-choice*.ti,ab. | 6634 |
| 40 | exp Decision Making/ and (patient* or user* or men or women).ti. | 88598 |
| 41 | exp Decision Support Systems, Clinical/ or exp Decision Support Techniques/ | 51406 |
| 42 | (health and utilit*).ti. | 3522 |
| 43 | gamble*.ti,ab. | 7193 |
| 44 | prospect theory.ti,ab. | 515 |
| 45 | preference score*.ti,ab. | 989 |
| 46 | preference elicitation.ti,ab. | 532 |
| 47 | health utilit*.ti,ab. | 5552 |
| 48 | utility value*.ti,ab. | 5402 |
| 49 | utility score*.ti,ab. | 4297 |
| 50 | utility estimate*.ti,ab. | 745 |
| 51 | health state.ti,ab. | 10671 |
| 52 | feeling thermometer*.ti,ab. | 119 |
| 53 | best-worst scaling.ti,ab. | 746 |
| 54 | time trade-off.ti,ab. | 2376 |
| 55 | TTO.ti,ab. | 2871 |
| 56 | probability trade-off.ti,ab. | 29 |
| 57 | preference based.ti,ab. | 3021 |
| 58 | multiattribute.ti,ab. | 500 |
| 59 | multi attribute.ti,ab. | 1413 |
| 60 | EuroQol 5D.ti,ab. | 3534 |
| 61 | EuroQol5D.ti,ab. | 67 |
| 62 | EQ5D.ti,ab. | 3840 |
| 63 | EQ 5D.ti,ab. | 32955 |
| 64 | SF6D.ti,ab. | 147 |
| 65 | SF 6D.ti,ab. | 1882 |
| 66 | HUI.ti,ab. | 3473 |
| 67 | 15D.ti,ab. | 3311 |
| 68 | (patient* adj (value* or satisfact*)).ti,ab. | 109391 |
| 69 | or/1-68 | 3424282 |
| 70 | immune deficiency/ | 103032 |
| 71 | (primary adj3 (immunodeficienc* or deficienc*)).mp. | 21188 |
| 72 | exp common variable immunodeficiency/ | 7248 |
| 73 | (common variable immunodeficiency or CVID).mp. | 8725 |
| 74 | exp chronic granulomatous disease/ | 7038 |
| 75 | (chronic granulomatous disease or CGD).mp. | 9474 |
| 76 | exp severe combined immunodeficiency/ | 9249 |
| 77 | severe combined immunodeficienc*.mp. | 12137 |
| 78 | exp hemophagocytic syndrome/ | 21656 |
| 79 | hemophagocytic lymphohistiocytosis.mp. | 9860 |
| 80 | ((IVIG or intravenous immunoglobulin) adj3 replace*).mp. | 838 |
| 81 | (antibod* adj3 deficienc*).mp. | 4482 |
| 82 | hyper IgM syndrome.mp. | 1480 |
| 83 | (inborn error* adj3 immunity).mp. | 3409 |
| 84 | or/70-83 | 158666 |
| 85 | 69 and 84 | 8645 |
| **PsycINFO** | | |
| **Database(s): APA PsycInfo <1806 to June 2026 Week 1>** | | |
| **Search Strategy:** | | **Number of Records** |
| 1 | exp Patient Participation/ | 3916 |
| 2 | preference*.ti,ab. | 127047 |
| 3 | (choice or choices).ti. | 31923 |
| 4 | value*.ti. | 42018 |
| 5 | health state values.ti,ab. | 99 |
| 6 | valuation*.ti. | 1311 |
| 7 | expectation*.ti,ab. | 108352 |
| 8 | attitude*.ti,ab. | 256110 |
| 9 | acceptab*.ti,ab. | 60520 |
| 10 | knowledge.ti,ab. | 403454 |
| 11 | point of view.ti,ab. | 23578 |
| 12 | user participation.ti,ab. | 419 |
| 13 | users participation.ti,ab. | 76 |
| 14 | patient participation.ti,ab. | 1086 |
| 15 | patients participation.ti,ab. | 334 |
| 16 | patient perspective*.ti,ab. | 1708 |
| 17 | patients perspective*.ti,ab. | 2628 |
| 18 | user perspective*.ti,ab. | 606 |
| 19 | users perspective*.ti,ab. | 565 |
| 20 | patient perce*.ti,ab. | 1824 |
| 21 | patients perce*.ti,ab. | 4495 |
| 22 | health perception*.ti,ab. | 1674 |
| 23 | user perce*.ti,ab. | 780 |
| 24 | users perce*.ti,ab. | 1598 |
| 25 | user view*.ti,ab. | 120 |
| 26 | users view*.ti,ab. | 380 |
| 27 | patient?? view*.ti,ab. | 1462 |
| 28 | ((decision* and mak*).ti. or decision mak*.ti,ab. or decisions mak*.ti,ab.) and (patient* or user* or men or women).ti,ab. | 32505 |
| 29 | user?? perspective*.ti,ab. | 1137 |
| 30 | user?? perce*.ti,ab. | 2284 |
| 31 | discrete choice*.ti,ab. | 1909 |
| 32 | decision board*.ti,ab. | 16 |
| 33 | decision analy*.ti,ab. | 1371 |
| 34 | decision-support.ti,ab. | 4592 |
| 35 | decision tool*.ti,ab. | 260 |
| 36 | decision aid*.ti,ab. | 1777 |
| 37 | discrete-choice*.ti,ab. | 1909 |
| 38 | exp Decision Making/ and (patient* or user* or men or women).ti. | 10388 |
| 39 | (health and utilit*).ti. | 722 |
| 40 | gamble*.ti,ab. | 6767 |
| 41 | prospect theory.ti,ab. | 1290 |
| 42 | preference score*.ti,ab. | 475 |
| 43 | preference elicitation.ti,ab. | 219 |
| 44 | health utilit*.ti,ab. | 771 |
| 45 | utility value*.ti,ab. | 941 |
| 46 | utility score*.ti,ab. | 544 |
| 47 | utility estimate*.ti,ab. | 129 |
| 48 | health state.ti,ab. | 1426 |
| 49 | feeling thermometer*.ti,ab. | 86 |
| 50 | best-worst scaling.ti,ab. | 209 |
| 51 | time trade-off.ti,ab. | 411 |
| 52 | TTO.ti,ab. | 285 |
| 53 | probability trade-off.ti,ab. | 6 |
| 54 | preference based.ti,ab. | 993 |
| 55 | multiattribute.ti,ab. | 588 |
| 56 | multi attribute.ti,ab. | 790 |
| 57 | EuroQol 5D.ti,ab. | 283 |
| 58 | EuroQol5D.ti,ab. | 2 |
| 59 | EQ5D.ti,ab. | 132 |
| 60 | EQ5D.ti,ab. | 132 |
| 61 | SF6D.ti,ab. | 16 |
| 62 | SF 6D.ti,ab. | 381 |
| 63 | HUI.ti,ab. | 634 |
| 64 | 15D.ti,ab. | 208 |
| 65 | (patient* adj (value* or satisfact*)).ti,ab. | 6913 |
| 66 | or/1-65 | 997389 |
| 67 | (primary adj3 (immunodeficienc* or deficienc*)).mp. | 203 |
| 68 | (common variable immunodeficiency or CVID).mp. | 25 |
| 69 | (chronic granulomatous disease or CGD).mp. | 30 |
| 70 | severe combined immunodeficienc*.mp. | 45 |
| 71 | hemophagocytic lymphohistiocytosis.mp. | 22 |
| 72 | ((IVIG or intravenous immunoglobulin) adj3 replace*).mp. | 2 |
| 73 | (antibod* adj3 deficienc*).mp. | 17 |
| 74 | hyper IgM syndrome.mp. | 2 |
| 75 | (inborn error* adj3 immunity).mp. | 5 |
| 76 | or/67-75 | 336 |
| 77 | 66 and 76 | 41 |
| **CINAHL** | | |
| **Search Strategy:** | | **Number of Records** |
| S1 | ((patient* N2 (attitude* OR decision* OR needs*)) OR (patient* N3 (prefer* OR participat* OR involve* OR perspective* OR view* OR activat* OR empower* OR collaborate)) OR (MH "Attitude to Health") OR (MH "Health Beliefs") OR (MH "Patient Satisfaction") OR (MH "Attitude to Risk") OR (MH "Consumer Participation")) AND ((MH "Primary Immunodeficiency Diseases+") OR (MH "Primary Immunodeficiency Diseases+") OR (MH "Common Variable Immunodeficiency") OR (MH "Severe Combined Immunodeficiency") OR TX (CVID OR CGD OR IVIG OR intravenous immunoglobulin OR inborn error)) | 294 |

**Supplementary Material 3.** Example transformation of quantitative data into qualitative data using critical meta-narrative synthesis with systematic profiles.

Below we provide an example of how we “qualitized” relevant quantitative data into qualitative data. We will use Machado 2024 [1] as our test case. This study analyzed surveys and interviews from 14 patients with primary hemophagocytic lymphohistiocytosis (HLH), with 9 (64%) patients reporting the lack of awareness of HLH and 8 (57%) patients reporting the lack of specialist knowledge on the signs/symptoms of HLH as the most common causes of frustration in the diagnosis journey and delays in getting a diagnosis.

1. Data and descriptive results were extracted into our standardized extraction with one cell for each quantitative data point.
2. Using a critical meta-narrative synthesis approach with systematic profiles (for this paper, “modal”, “average”, and “comparative” approaches) [2], we created an adjacent column to the data point and converted the quantitative data point to a narrative comment. Considering the reported data and the original study’s conclusions, the following narrative summary was derived using a modal profile:
   a. “A majority of patients and caregivers cited lack of awareness of HLH, lack of specialist knowledge on the signs/symptoms of HLH, and lack of awareness of HLH by doctors as the most common causes of frustration in their diagnosis journey.”
3. This process was repeated for each quantitative study.

References:

1. Machado T, Lawrence J, Eriksson D, Donohue J. A need for greater awareness: a mixed methods study of patient and healthcare professional perspectives on the diagnosis journey for haemophagocytic lymphohistiocytosis (HLH). Hematology. 2024 Dec;29(1):2358261. doi: 10.1080/16078454.2024.2358261.
2. Karimi M, Clark AM. How do patients' values influence heart failure self-care decision-making?: A mixed-methods systematic review. Int J Nurs Stud. 2016 Jul;59:89-104. doi: 10.1016/j.ijnurstu.2016.03.010.

**Supplementary Material 4**. Risk of bias assessments for the included qualitative studies.

| **Study** | **Was there a clear statement of the aims of the research?** | **Is the methodology appropriate?** | **Was the research design appropriate to address the aims of the research?** | **Was the recruitment strategy appropriate to the aims of the research?** | **Was the data collected in a way that addressed the research issue?** | **Has the relationship between researcher and participants been adequately considered?** | **Have ethical issues been taken into consideration?** | **Was the data analysis sufficiently rigorous?** | **Is there a clear statement of findings?** | **How valuable is the research?** |
| --- | --- | --- | --- | --- | --- | --- | --- | --- | --- | --- |
| AhmadAzahari 2023 | Yes | Yes | Yes | Yes | Yes | Yes | Yes | Yes | Yes | Yes |
| AhmedMeelad 2022 | Yes | Yes | Yes | Yes | Yes | Yes | Yes | Yes | Yes | Yes |
| Beers 2025 | Yes | Yes | Yes | Yes | Yes | Yes | Yes | Yes | Yes | Yes |
| Clark 2025 | Yes | Yes | Yes | Yes | Yes | Can't Tell | Yes | Yes | Yes | Yes |
| Cozon 2018 | Yes | Yes | Yes | Yes | Yes | Yes | Yes | No | Yes | Yes |
| Davidson 2024 | Yes | Yes | Yes | Yes | Yes | Yes | Yes | Yes | Yes | Yes |
| Franzese 2025 | Yes | Yes | Yes | Yes | Yes | Can't Tell | Yes | Yes | Yes | Yes |
| Gardulf 1995 | Yes | No | No | No | Yes | Yes | Yes | No | Yes | Yes |
| Hansen 2004 | Yes | No | No | No | Yes | Yes | Yes | No | Yes | Yes |
| Janssen 2021 | Yes | Yes | Yes | Yes | Yes | Yes | Yes | Yes | Yes | Yes |
| Johansen 2026 | Yes | Yes | Yes | Yes | Yes | Yes | Yes | Yes | Yes | Yes |
| Jones 2020b | Yes | Yes | Yes | Yes | Yes | Yes | Yes | Yes | Yes | Yes |
| Joseph 2016 | Yes | Yes | Yes | Yes | Yes | Can't Tell | Yes | No | Yes | Yes |
| Kutsa 2022 | Yes | Yes | Yes | Yes | Yes | Can't Tell | Yes | Yes | Yes | Yes |
| Margolis 2017 | Yes | Yes | Yes | Yes | Yes | Can't Tell | Yes | Yes | Yes | Yes |
| Ouimet 2023 | Yes | Yes | Yes | Yes | Yes | Can't Tell | Yes | Yes | Yes | Yes |
| Ponsford 2015 | Yes | No | No | No | Yes | Yes | Yes | No | Yes | Yes |
| Pursey 2025 | Yes | Yes | Yes | Yes | Yes | Yes | Yes | Yes | Yes | Yes |
| Raspa 2020 | Yes | Yes | Yes | Yes | Yes | Can't Tell | Yes | No | Yes | Yes |
| Smith 2021 | Yes | No | No | No | Yes | Yes | Yes | No | Yes | Yes |
| Sultan 2017 | Yes | No | No | No | Yes | Yes | Yes | No | Yes | Yes |
| Wolf 2026 | Yes | Yes | Yes | Yes | Yes | Can't Tell | Yes | Yes | Yes | Yes |

**Supplementary Material 5**. Risk of bias assessments for the included quantitative studies.

| **Study** | **Was an appropriate study sample selected from the sampling frame?** | **Was the attrition sufficiently low to minimize the risk of bias?** | **Was the instrument used for eliciting relative importance of outcomes valid and reliable?** | **Was the instrument administered in the intended way?** | **Was a valid representation of the outcome (health state) utilized?** | **Did the researchers check the understanding of the instrument?** | **Were the results analyzed appropriately to avoid influence of bias and confounding?** |
| --- | --- | --- | --- | --- | --- | --- | --- |
| Aberumand 2022 | Probably Yes | Probably Yes | Probably Yes | Probably Yes | Not Applicable | No | Yes |
| Agnihotri 2021 | Probably Yes | Probably No | Probably Yes | Probably Yes | Not Applicable | No | Yes |
| Allen 2022 | Probably Yes | Probably No | Probably Yes | Probably Yes | Not Applicable | No | Yes |
| Chatelier 2014 | Probably Yes | Yes | Probably Yes | Probably Yes | Not Applicable | No | Yes |
| Duff 2014 | Yes | Probably No | Probably Yes | Probably Yes | Not Applicable | No | Yes |
| Eltan 2022 | Yes | Probably No | Probably Yes | Probably Yes | Not Applicable | No | Yes |
| Espanol 2012 | Probably Yes | Probably No | Probably Yes | Probably Yes | Not Applicable | No | Yes |
| Espanol 2014 | Yes | Probably No | Probably Yes | Probably Yes | Not Applicable | No | Yes |
| Gardulf 1995 | Yes | Yes | Probably Yes | Probably Yes | Not Applicable | No | Yes |
| Gardulf 2004 | Yes | Probably No | Probably Yes | Probably Yes | Not Applicable | No | Yes |
| Geoffroy 2025 | Probably Yes | Probably No | Probably Yes | Probably Yes | Not Applicable | No | Yes |
| Gonzalez 2022 | Yes | Probably No | Yes | Yes | Yes | Yes | Yes |
| Hansen 2004 | Yes | Probably No | Probably Yes | Probably Yes | Not Applicable | No | Yes |
| Ito 2014 | Probably Yes | Probably No | Probably Yes | Probably Yes | Not Applicable | No | Yes |
| Jones 2020a | Yes | Probably Yes | Probably Yes | Probably Yes | Not Applicable | No | Yes |
| Karali 2025 | Yes | Probably No | Probably Yes | Probably Yes | Not Applicable | No | Yes |
| Keestra 1988 | Yes | Probably No | Probably Yes | Probably Yes | Not Applicable | No | Yes |
| Lechanska-Helman 2020 | Yes | Probably No | Probably Yes | Probably Yes | Not Applicable | No | Yes |
| Machado 2024 | Yes | Probably No | Probably Yes | Probably Yes | Not Applicable | No | Yes |
| Meckley 2020 | Yes | Probably Yes | Probably Yes | Probably Yes | Not Applicable | No | Yes |
| Mohamed 2012 | Yes | Probably No | Probably Yes | Yes | Probably Yes | Yes | Yes |
| Myers 2025 | Probably Yes | Probably Yes | Probably Yes | Probably Yes | Probably Yes | Probably Yes | Probably Yes |
| NapiorkowskaBaran 2021 | Yes | Probably No | Probably Yes | Probably Yes | Not Applicable | No | Yes |
| Pergent 2023 | Yes | Probably No | Probably Yes | Probably Yes | Not Applicable | No | Yes |
| Raspa 2020 | Yes | Probably No | Probably Yes | Probably Yes | Not Applicable | No | Yes |
| Reid 2014 | Yes | Probably Yes | Probably Yes | Probably Yes | Not Applicable | No | Yes |
| Runken 2016 | Probably Yes | Probably No | Probably Yes | Probably Yes | Not Applicable | No | Yes |
| Runken 2017 | Probably Yes | Probably No | Probably Yes | Probably Yes | Not Applicable | No | Yes |
| Rutland 2025 | Yes | Probably No | Probably Yes | Probably Yes | Not Applicable | No | Yes |
| Schwaneck 2025 | Yes | Probably No | Probably Yes | Probably Yes | Not Applicable | No | Yes |
| Sowers 2022 | Yes | Probably No | Probably Yes | Probably Yes | Not Applicable | No | Yes |
| Suleymanova 2026 | Yes | Probably No | Probably Yes | Probably Yes | Not Applicable | No | Yes |
| Sultan 2017 | Yes | Yes | Probably Yes | Probably Yes | Not Applicable | No | Yes |
| Wasserman 2017 | Probably Yes | Probably No | Probably Yes | Probably Yes | Not Applicable | No | Yes |

**Supplementary Material 6.** Thematic synthesis for review theme of “Patients and caregivers are more willing to engage with and accept treatment if counseled by HCPs knowledgeable in IEI.”

| **Study** | **Data Type** | **Perspective** | **Quote or Qualitized Narrative Summary** | **Code** | **Descriptive Theme** | **Overall Analytical Theme** |
| --- | --- | --- | --- | --- | --- | --- |
| Agnihotri 2021 | Quantitative | Patient/Caregiver | A majority of caregivers and patients believe that providers should prepare IEI patients for transition around ages 15-17 | Desire for more knowledge and resources regarding IEI | Preference for care and counseling from HCPs knowledgeable about IEI diagnosis, management, and resources | Patients and caregivers are more willing to engage with and accept treatment if counseled by HCPs knowledgeable in IEI |
| Agnihotri 2021 | Quantitative | Patient/Caregiver | All caregivers and most patients agree that during transition patients should be educated about medications, patient condition, and symptoms that require seeking health care | Desire for more knowledge and resources regarding IEI |  |  |
| Agnihotri 2021 | Quantitative | Patient/Caregiver | Most caregivers and almost all patients prefer a transition coordinator for pediatric to adult care | Desire for more knowledge and resources regarding IEI |  |  |
| AhmadAzahari 2023 | Qualitative | Caregiver | …public awareness on genetic testing is very low, moreover, for Malay community they believe in superstition saying that we did something wrong. | Desire for more knowledge and resources regarding IEI |  |  |
| AhmadAzahari 2023 | Qualitative | Caregiver | …when I first heard about genetic testing, I also don’t know what it is actually. | Desire for more knowledge and resources regarding IEI |  |  |
| AhmadAzahari 2023 | Qualitative | Caregiver | …but I find it difficult to understand (genetic test and PID), so I keep asking the doctors and they say that if I want more information, I should also try Googling it. | Desire for more knowledge and resources regarding IEI |  |  |
| AhmadAzahari 2023 | Qualitative | Caregiver | …I am not aware about the cost and was not informed about it either (cost for genetic testing). | Desire for more knowledge and resources regarding IEI |  |  |
| AhmadAzahari 2023 | Qualitative | Caregiver | …I prefer both ways, whether it’s obtaining information online or through doctors. | Desire for more knowledge and resources regarding IEI |  |  |
| AhmadAzahari 2023 | Qualitative | Caregiver | …Initially, we relied solely on the doctors for information, but at that time, we didn’t have internet access. So, we turned to our friends who had access to Google and printed out the information (genetic disease) for us. | Desire for more knowledge and resources regarding IEI |  |  |
| AhmedMeelad 2022 | Qualitative | Caregiver | Before that I haven't event met anyone from the immunologist team, they just gave me the name of the disease, the category. So then I looked it up. Then I got more confused than before, and I started to think of getting on ways of how to get him out from the hospital. | Desire for more knowledge and resources regarding IEI |  |  |
| AhmedMeelad 2022 | Qualitative | Caregiver | But if we have the kind of society that lacks awareness and knowledge or we are surrounded with those kinds of people that does not have the awareness about this disease. | Desire for more knowledge and resources regarding IEI |  |  |
| AhmedMeelad 2022 | Qualitative | Caregiver | Previously I only knew the type like XXX. When I entered MyPOPI, it was then I got to know that there are other types of PID that I haven't even heard about | Desire for more knowledge and resources regarding IEI |  |  |
| Allen 2022 | Quantitative | Patient | A majority of patients are interested in receiving more information on adjusting to life post-treatment | Desire for more knowledge and resources regarding IEI |  |  |
| Allen 2022 | Quantitative | Patient | A majority of patients are interested in receiving more information on returning to school/work | Desire for more knowledge and resources regarding IEI |  |  |
| Allen 2022 | Quantitative | Patient | A minority of patients are interested in receiving more information nutrition and healthy eating | Desire for more knowledge and resources regarding IEI |  |  |
| Allen 2022 | Quantitative | Patient | A minority of patients are interested in receiving more information on adjusting to diagnosis and treatment | Desire for more knowledge and resources regarding IEI |  |  |
| Beers 2025 | Qualitative | Patient | “I’ve also learned a lot of the medical terminology for my issues; that makes me feel a little bit more in control whenever I’m talking to a doctor.” | Desire for more knowledge and resources regarding IEI |  |  |
| Beers 2025 | Qualitative | Patient | “If you know more about your illness, you can live your life better. … If you don't know much about your illness, you keep struggling and keep asking, ‘Why is this happening this to me? What is happening? Why do I get all those infections?’” | Desire for more knowledge and resources regarding IEI |  |  |
| Clark 2025 | Qualitative | Caregiver | “A sourcing of information about your condition that is correct and just look here for what you need, that would be helpful…and we did get that to an extent, but something as simple as what’s the life expectancy? I don’t think we got that. I was finding that out from medical journal… but because you have these kinds of questions, which then you spiral…and you just end up Googling…” | Desire for more knowledge and resources regarding IEI |  |  |
| Clark 2025 | Qualitative | Caregiver | “I think the hardest thing is the uncertainty with the whole thing. There are no answers, and you have no idea of the outcome.” | Desire for more knowledge and resources regarding IEI |  |  |
| Clark 2025 | Qualitative | Caregiver | “We’re really happy with really with our doctors, but they don’t necessarily explain things to the kids well… no one’s really ever explained to him what’s going on.” | Desire for more knowledge and resources regarding IEI |  |  |
| Davidson 2024 | Qualitative | Patient | [E]very time we’d go to a doctor, it would be “Well, do you think this is related?” and you’d hear ‘Oh, well, maybe.’ ‘Oh, I don’t think so,’ and then you’d get other doctors who are like ‘Yeah, I’m 100% sure it’s related,’ and now, they’re pretty well sure it’s related. So, when we got this [PIK3CD GOF disorder] diagnosis … It started terrifying me because none of [my brother’s] symptoms were the same as my symptoms. So, I was wonder[ing] if it was related to the mutation and it made me fear for my kids. | Desire for more knowledge and resources regarding IEI |  |  |
| Gardulf 1995 | Quantitative | Patient | Patients generally desired for more knowledge about their antibody deficiency | Desire for more knowledge and resources regarding IEI |  |  |
| Jones 2020b | Qualitative | Patient | “I thought it was a quick-fix thing. Obviously, what I didn’t hear, which I’ve now got the grip of, it has been explained to me, that of course, it isn’t is it? It’s long-term, well it’s for life basically” | Desire for more knowledge and resources regarding IEI |  |  |
| Joseph 2016 | Qualitative | Caregiver | “I think personally, as a newborn parent, I had no idea of a lot of things and I think it’s great that it’s mandatory because you’re not aware of diseases and things that babies are diagnosed with. …we all have kids with medical issues, but I think that before I had my children [if] they had asked me, ‘Do you want [newborn screening]?’ No, I wouldn't want them to be tested. Now I would answer, absolutely ‘Yeah, test’.” | Desire for more knowledge and resources regarding IEI |  |  |
| Kutsa 2022 | Qualitative | Caregiver | So what we did was we made a kind of a pro/con chart, you know, we sat down and we wrote down, I wrote out all the different treatment options and, you know, what the positives and negatives are of each, which ones would need chemotherapy, which ones wouldn’t, and what possible side effects could happen and stuff like that. And that was a really, really important tool for us to help make the decisions. | Desire for more knowledge and resources regarding IEI |  |  |
| Kutsa 2022 | Qualitative | Caregiver | We just had to have faith in his doctors and ask a lot of questions. They were the ones that always calmed us down and told us, like, ‘this is our plan. He’s going to be okay. We really think that this is going to work.’ We really just had to listen to them | Desire for more knowledge and resources regarding IEI |  |  |
| Margolis 2017 | Qualitative | Patient | "Have a conference at NIH for adolescents with CGD." | Desire for more knowledge and resources regarding IEI |  |  |
| Margolis 2017 | Qualitative | Patient | “[The doctor] pesters me and she’s persistent and I don’t want to disappoint her. I finally got myself organized. The doctors motivated me in the past few years and I started taking care of things myself.” | Desire for more knowledge and resources regarding IEI |  |  |
| Margolis 2017 | Qualitative | Patient | “A pamphlet outlining what goes into a visit starting with travel, reservations, clinic appointment.” | Desire for more knowledge and resources regarding IEI |  |  |
| Margolis 2017 | Qualitative | Patient | “Do more walk-throughs on the unit to get familiar with the unit and nurses.” | Desire for more knowledge and resources regarding IEI |  |  |
| Margolis 2017 | Qualitative | Patient | “Encourage patients to be an active participant. It’s important to know what and why.” | Desire for more knowledge and resources regarding IEI |  |  |
| Margolis 2017 | Qualitative | Patient | “Have the doctor tell parents to talk to their kids at age 15 or 16.” | Desire for more knowledge and resources regarding IEI |  |  |
| Margolis 2017 | Qualitative | Patient | “Make sure the patient is comfortable in the setting they are in. They need to (1) understand the importance of taking medicine, (2) if getting sick contact the doctor right away. (3) Understanding the disease is highly important and (4) Know it is possible to live a full healthy lifestyle if staying on top of what they need to know.” | Desire for more knowledge and resources regarding IEI |  |  |
| Margolis 2017 | Qualitative | Patient | “My parents always encouraged me to ask questions. The younger the better. My mom got scolded by the doctors for not taking a more active role but she said she wasn’t always going to be around.” | Desire for more knowledge and resources regarding IEI |  |  |
| Margolis 2017 | Qualitative | Patient | “Sit down one on one with your doctor to talk about your meds and disease.” | Desire for more knowledge and resources regarding IEI |  |  |
| Margolis 2017 | Qualitative | Patient | “Start young, like around 15 or 16, a few years before. Talk about their flights and appointments with their parents there but not doing it for them.” | Desire for more knowledge and resources regarding IEI |  |  |
| Ouimet 2023 | Qualitative | Caregiver | “But that too, the studies on this syndrome, I have books, I even ordered a book from the United States, it is until adulthood. But once it concerns adulthood, there’s nothing left.” | Desire for more knowledge and resources regarding IEI |  |  |
| Ouimet 2023 | Qualitative | Caregiver | “didn’t know that once at the adult hospital, that he [her youth] wasn’t going to have a [hospital] room for himself [ … ] We didn’t know, I don’t think even my husband knew.” | Desire for more knowledge and resources regarding IEI |  |  |
| Ouimet 2023 | Qualitative | Caregiver | Some participants did not know that the transition occurred at 18 years old and thought that “in certain cases [the pediatric] hospital kept the child until 21 years old”. As this caregiver further explained: “It’s weird, we knew 18 was the adult age, but we thought since our daughter has a mental age of seven, eight or whatever, that they would keep her for her exceptional conditions. But no.” | Desire for more knowledge and resources regarding IEI |  |  |
| Raspa 2020 | Qualitative | Caregiver | “Throughout the journey, the bone marrow transplant makes you feel so helpless. If there is no donor what do you do? You just keep waiting. I feel like a lot of education work needs to be done in that area.” | Desire for more knowledge and resources regarding IEI |  |  |
| Raspa 2020 | Quantitative | Caregiver | Caregivers generally desire additional information regarding the screening process, expectations for their child's condition and management, treatment options and their side effects, access to specialists, ways to optimize their child's wellbeing, where to seek financial assistance, risk of having another child with an IEI, how to educate family and friends about their child's condition, and how to education future providers about their child's condition | Desire for more knowledge and resources regarding IEI |  |  |
| Reid 2014 | Quantitative | Patient/Caregiver | Patients and caregivers generally ranked a lack of supervision as the primary concern related to switching to home-based SCIg, followed by costs, frequent injections, lost time, having to poke themselves, and supply storage | Desire for more knowledge and resources regarding IEI |  |  |
| Smith 2021 | Qualitative | Caregiver | “I really just remember praying he would survive. But once he was through the transplant, I was expecting him not to need IVIGb and that was not the case.” | Desire for more knowledge and resources regarding IEI |  |  |
| Smith 2021 | Qualitative | Caregiver | “Our little one has normal counts of B-cells so we assumed he wouldn’t need SCIGc, now we are finding out it is because all those B-cells are his and don’t function properly because of the SCID, that he will need IgG replacement for life.” | Desire for more knowledge and resources regarding IEI |  |  |
| Wasserman 2017 | Quantitative | Patient | Some patients felt their provider explained the different routes of administration in a hurried, unclear or confusing manner, and a minority of patients felt that the routes of administration decision was made jointly between the patient and physician | Desire for more knowledge and resources regarding IEI |  |  |
| AhmadAzahari 2023 | Qualitative | Caregiver | …but it was his (child) of luck when we get to meet the right doctor who specialises in that specific field (PID)… | Preference for HCPs knowledgeable about IEI |  |  |
| AhmadAzahari 2023 | Qualitative | Caregiver | …even in the hospital if we tell them about SCID, they don’t understand, then who are we to explain to the specialist…imagine O&G specialist told me that SCID was not a genetic disease. | Preference for HCPs knowledgeable about IEI |  |  |
| AhmadAzahari 2023 | Qualitative | Caregiver | …I think the awareness with the doctors is important. | Preference for HCPs knowledgeable about IEI |  |  |
| AhmadAzahari 2023 | Qualitative | Caregiver | During my first child, I felt that it took a long time to confirm and detect the disease (SCID). But according to the doctor in the Alor Setar, Kedah area, they mentioned that now there are new specialists who are focusing on PID, specifically the disease related to PID. | Preference for HCPs knowledgeable about IEI |  |  |
| Franzese 2025 | Qualitative | Patient | “They actually sent me out a nurse who had never used the pump before. And I basically knew more about it than she did. She was actually Googling how to use the pump. And so that was quite discouraging.” | Preference for HCPs knowledgeable about IEI |  |  |
| Hansen 2004 | Quantitative | Patient | All patients perceived physicians involved with their prenatal care to have insufficient knowledge about PAD or IgRT and most patients perceived midwives to have insufficient knowledge about PAD or IgRT | Preference for HCPs knowledgeable about IEI |  |  |
| Hansen 2004 | Qualitative | Patient | I always feel a little bit silly when I have to explain about my antibody deficiency, as if it is not a “real” disease. I wish the midwives and the physicians at the prenatal clinics had known more about antibody deficiencies. | Preference for HCPs knowledgeable about IEI |  |  |
| Hansen 2004 | Qualitative | Patient | I wish the midwives had been less worried all the time and that the midwives and the physicians had listened to me and to my knowledge of my disease. | Preference for HCPs knowledgeable about IEI |  |  |
| Hansen 2004 | Qualitative | Patient | No one knew anything about the IgG therapy and when I tried to get the physicians to understand that I don’t react like others do when I get an infection and am put on “weak” antibiotics, my information was ignored. | Preference for HCPs knowledgeable about IEI |  |  |
| Hansen 2004 | Qualitative | Patient | The physician said that I should not continue with the IgG therapy now that I was pregnant. | Preference for HCPs knowledgeable about IEI |  |  |
| Hansen 2004 | Qualitative | Patient | They thought I had AIDS when they read my medical record. They were very incompetent. | Preference for HCPs knowledgeable about IEI |  |  |
| Janssen 2021 | Qualitative | Patient | The stupid thing is that there is a pulmonology guideline for recurrent airway infections. According to the protocol he had to make a referral. He didn’t know that but then at least be honest enough to say that you don’t know. | Preference for HCPs knowledgeable about IEI |  |  |
| Keestra 1988 | Quantitative | Patient | A majority of patients consider a specialist at an academic center to be the most trusted physician for their condition | Preference for HCPs knowledgeable about IEI |  |  |
| Kutsa 2022 | Qualitative | Caregiver | And then when they found out when the first transplant didn’t work, I said, let’s look at [child’s brother as a donor], you know, to see if he’s a match. And they didn’t want to do that [due to concerns about sibling’s size and age]. They said, ‘We can’t do it. Let’s do the stem cell [referring to HSCT with cord blood that was 5 out of 6 match].’ So, I had to push, I had to ask questions and keep looking. And I saw a status up on the Facebook group, and that kind of directed me and sent me to the doctors that had the experience that said, ‘yes, [sibling] could be the donor.’ There’s a lot of success with cord blood. But since my [child] had a better alternative [the sibling], and [the doctor she found via the Facebook group] said it could be done…that’s where everything went successful. | Preference for HCPs knowledgeable about IEI |  |  |
| Kutsa 2022 | Qualitative | Caregiver | When we [the child] were diagnosed, we were sent to [Hospital Name] in [City]. And they had a wonderful team there and very knowledgeable, you know, they were answering all our questions and help put our mind at ease. But it was so new to us that, well, like it is probably to most families. | Preference for HCPs knowledgeable about IEI |  |  |
| Machado 2024 | Quantitative | Patient/Caregiver | A majority of patients and caregivers cited lack of awareness of HLH, lack of specialist knowledge on the signs/symptoms of HLH, and lack of awareness of HLH by doctors as the most common causes of frustration in their diagnosis journey | Preference for HCPs knowledgeable about IEI |  |  |
| Ouimet 2023 | Qualitative | Caregiver | “[ … ] it kind of threw me for a loop when the coordinator told me … Because I said, ‘Do you have any doctors to suggest afterwards?’ She said, ‘No, now you’re falling into a pfft.’ When I told her ‘Doctors who know about velocardiofacial syndrome’ she said, ‘Good luck.” So that’s when I went ‘oh boy. Oh boy, okay. [ … ] That’s worrisome.’” | Preference for HCPs knowledgeable about IEI |  |  |
| Ouimet 2023 | Qualitative | Caregiver | “…when my daughter is sick, no matter what I say, no one understands what I’m saying, it’s like I’m speaking another language! It’s fun to be in places where people understand what you’re going through. You can go consult anywhere for cancer, but for PANDAS, you can’t go anywhere.” | Preference for HCPs knowledgeable about IEI |  |  |
| Ouimet 2023 | Qualitative | Patient | “For sure, it was like a little bit of an upheaval to know that basically I didn’t have the right diagnosis and that all the therapies that I had done before and the medication and everything, it’s kind of like I did them for nothing.” | Preference for HCPs knowledgeable about IEI |  |  |
| Ouimet 2023 | Qualitative | Patient | “I know my situation; I’ve been living with myself for 21 years. I came in with a mouth infection. They wanted to give me something because I couldn’t walk too much, I had a big arthritis crisis, they gave me fentanyl, morphine, Dilaudid. Dilaudid thins the blood. I have a blood factor that is complicated, you can’t give me a blood thinner. If I fall, I cut myself or whatever, I bleed out. So, I tell them. He says, “Well anyway, that’s not what we were ordered to do.” I said, “Fine, but I’m not taking your pill. You can write in the file that I refuse it.” So you know, you have to fight constantly, constantly. Like I tell them I’m allergic to latex. “Ah it’s not a big deal.” They put on latex gloves. It’s because I become swollen all over. You know your situation, but they’re not listening to you at all.” | Preference for HCPs knowledgeable about IEI |  |  |
| Ouimet 2023 | Qualitative | Caregiver | “The doctors really delayed prescribing medication to him because they stayed there just to observe him. They had observed him for two months without giving him anything except some kind of painkillers and that’s it. But not to cure, it’s just to control, calm, but not to cure. The disease kept developing during that time. [ … ] I think it left him with a lot of sequelae.” | Preference for HCPs knowledgeable about IEI |  |  |
| Reid 2014 | Quantitative | Patient/Caregiver | Recommendation from a clinical immunologist was related to more willingness to swap from hospital to home based IVIG | Preference for HCPs knowledgeable about IEI |  |  |
| Reid 2014 | Quantitative | Patient/Caregiver | Recommendation from a clinical immunologist was related to more willingness to swap from hospital to home based SCIg | Preference for HCPs knowledgeable about IEI |  |  |
| Reid 2014 | Quantitative | Patient/Caregiver | Recommendation from a family physician was related to more unwillingness to swap from hospital to home based IVIG | Preference for HCPs knowledgeable about IEI |  |  |
| Reid 2014 | Quantitative | Patient/Caregiver | Recommendation from a family physician was related to more unwillingness to swap from hospital to home based SCIg | Preference for HCPs knowledgeable about IEI |  |  |
| Wasserman 2017 | Quantitative | Patient | Some patients felt their provider explained the different routes of administration in a hurried, unclear or confusing manner, and a minority of patients felt that the routes of administration decision was made jointly between the patient and physician | Preference for HCPs knowledgeable about IEI |  |  |
| Wolf 2026 | Qualitative | Caregiver | “We were lucky to end up at a university center. Without that referral, we might still be waiting.” | Preference for HCPs knowledgeable about IEI |  |  |
| AhmadAzahari 2023 | Qualitative | Caregiver | During my first child, I felt that it took a long time to confirm and detect the disease (SCID). But according to the doctor in the Alor Setar, Kedah area, they mentioned that now there are new specialists who are focusing on PID, specifically the disease related to PID. | Concerns regarding delay/accuracy in diagnosis of IEI |  |  |
| AhmedMeelad 2022 | Qualitative | Caregiver | He also took medication for TB, but it did not work because it is not TB… I did not count those treatments as not helping but it's just at that point in time, the doctors had not found the real underlying cause of his problem. | Concerns regarding delay/accuracy in diagnosis of IEI |  |  |
| AhmedMeelad 2022 | Qualitative | Caregiver | It is like she has one, but the doctor did not properly diagnose her yet. | Concerns regarding delay/accuracy in diagnosis of IEI |  |  |
| Beers 2025 | Qualitative | Patient | “What I have still is just kind of like unknown, and I think that's what's the hardest part. … I've just accepted that whether it's got some acronym or not, it doesn't really matter.” | Concerns regarding delay/accuracy in diagnosis of IEI |  |  |
| Ouimet 2023 | Qualitative | Patient | “For sure, it was like a little bit of an upheaval to know that basically I didn’t have the right diagnosis and that all the therapies that I had done before and the medication and everything, it’s kind of like I did them for nothing.” | Concerns regarding delay/accuracy in diagnosis of IEI |  |  |
| Pursey 2025 | Qualitative | Caregiver | "I think I have a bit of trauma over not wanting anything else missed [after delayed diagnosis]” | Concerns regarding delay/accuracy in diagnosis of IEI |  |  |
| Pursey 2025 | Qualitative | Caregiver | "If we knew from newborn screening, we would have kept him isolated. Maybe he wouldn’t have gotten sick. Then you think what life would have been like.” | Concerns regarding delay/accuracy in diagnosis of IEI |  |  |
| Wolf 2026 | Qualitative | Caregiver | “We were lucky to end up at a university center. Without that referral, we might still be waiting.” | Concerns regarding delay/accuracy in diagnosis of IEI |  |  |
| Wolf 2026 | Qualitative | Caregiver | “We were in and out of hospitals for years. Nobody thought to check the immune system. Only when our new pediatrician ran more comprehensive tests did things move forward.” | Concerns regarding delay/accuracy in diagnosis of IEI |  |  |

**Supplementary Material 7.** Thematic synthesis for review theme of “Patients and caregivers place a high value on IEI treatments that minimize needle pricks and adverse effects.”

| **Study** | **Data Type** | **Perspective** | **Quote or Qualitized Narrative Summary** | **Code** | **Descriptive Theme** | **Overall Analytical Theme** |
| --- | --- | --- | --- | --- | --- | --- |
| Espanol 2012 | Quantitative | Patient/Caregiver | A minority of patients and caregivers reported therapy without needles/infusion as an ideal treatment | Preference for IgRT with fewer needle pricks | Preference for IEI treatments with minimal pain and discomfort | Patients and caregivers place a high value on IEI treatments that minimize needle pricks and adverse effects |
| Espanol 2014 | Quantitative | Patient/Caregiver | Patients and caregivers preferred fewer needle sticks during IgRT | Preference for IgRT with fewer needle pricks |  |  |
| Franzese 2025 | Qualitative | Patient | “It was pretty nerve-racking. You’re nervous because who wants to stick themselves with needles? But I knew that this treatment would be best for me and my family, and could sort of help me live a healthy life.” | Preference for IgRT with fewer needle pricks |  |  |
| Gardulf 1995 | Qualitative | Patient | Independently of the method, to prick myself with the needle is the worst thing | Preference for IgRT with fewer needle pricks |  |  |
| Gonzalez 2022 | Quantitative | Patient | Patients generally prefer IgRT treatment with fewer needle pricks | Preference for IgRT with fewer needle pricks |  |  |
| Jones 2020b | Qualitative | Patient | “… can’t get used to it no, never. I can’t look at it, you know when they’re putting it in, I can’t look at it at all” | Preference for IgRT with fewer needle pricks |  |  |
| Jones 2020b | Qualitative | Patient | “I said ‘no chance, I’m not doing that’. I won’t stick a needle- I can’t look at people stick needles in me. [Nurse], she persuaded me to have a go at doing it, which we did. It took 3 or 4 months, maybe longer.” | Preference for IgRT with fewer needle pricks |  |  |
| Jones 2020a | Quantitative | Patient | A majority of patients always, often, or sometimes found it painful when the needle is inserted for IgRT | Preference for IgRT with fewer needle pricks |  |  |
| Mohamed 2012 | Quantitative | Patient/Caregiver | Patients and caregivers generally prefer fewer needle sticks per IgRT interval | Preference for IgRT with fewer needle pricks |  |  |
| Ponsford 2015 | Qualitative | Patient | Keen to reduce frequency of needles (2 per week) | Preference for IgRT with fewer needle pricks |  |  |
| Ponsford 2015 | Qualitative | Patient | Needle phobia – missing infusions when SCIg given weekly. Always thinking about next infusion | Preference for IgRT with fewer needle pricks |  |  |
| Ponsford 2015 | Qualitative | Patient | Needle phobia – wanted to switch to reduce number of needles | Preference for IgRT with fewer needle pricks |  |  |
| Ponsford 2015 | Qualitative | Patient | Only one needle every 3 weeks | Preference for IgRT with fewer needle pricks |  |  |
| Sultan 2017 | Qualitative | Patient | "Having two needles in your stomach is not comfortable" | Preference for IgRT with fewer needle pricks |  |  |
| Sultan 2017 | Qualitative | Patient | "I’m always anxious because of the needle" | Preference for IgRT with fewer needle pricks |  |  |
| Sultan 2017 | Quantitative | Caregiver | Some caregivers report that that discomfort was a perceived issue of SCIg | Preference for IgRT with fewer needle pricks |  |  |
| Sultan 2017 | Quantitative | Patient | Some patients report that discomfort was a perceived issue of SCIg | Preference for IgRT with fewer needle pricks |  |  |
| Jones 2020b | Qualitative | Patient | “I get everything prepared and I do it, and it does help, visualising it in that way. I hate afterwards when I’ve finished, the site area is really sore, it’s swollen and because I’ve got a scar from my belly button down” | Preference for less painful treatments |  |  |
| Ponsford 2015 | Qualitative | Patient | Annoying doing every week although a great treatment. More painful, would last 24 h. Reminded me I was ill. More inconvenient, I had to take it with me when I travelled | Preference for less painful treatments |  |  |
| Ponsford 2015 | Qualitative | Patient | Really happy with this. Only painful for a few hours. Only infuse every 3 weeks now, so barely remember I have to do it. No need to take it with me when I travel | Preference for less painful treatments |  |  |
| Sultan 2017 | Qualitative | Patient | "Infusion sites can be painful for 24 hours" | Preference for less painful treatments |  |  |
| Sultan 2017 | Qualitative | Patient | "SCIg needles are less painful" | Preference for less painful treatments |  |  |
| Sultan 2017 | Quantitative | Patient | A majority of patients report that pain was a perceived issue of SCIg | Preference for less painful treatments |  |  |
| Sultan 2017 | Quantitative | Caregiver | A minority of caregivers report that pain was a perceived issue of SCIg | Preference for less painful treatments |  |  |
| Sultan 2017 | Quantitative | Patient | Some patients report that limited pain was a benefit of SCIg | Preference for less painful treatments |  |  |
| Eltan 2022 | Quantitative | Patient | A majority of patients reported that not having any side effects was a reason for being satisfied with SCIg | Preference for IEI treatments with fewer side effects | Preference for IEI treatments with fewer side effects |  |
| Eltan 2022 | Quantitative | Patient | A majority of patients reported that not having vascular problems was a reason for being satisfied with SCIg | Preference for IEI treatments with fewer side effects |  |  |
| Espanol 2012 | Quantitative | Patient/Caregiver | A minority of patients and caregivers reported treatment with fewer side effects as an ideal treatment | Preference for IEI treatments with fewer side effects |  |  |
| Gonzalez 2022 | Quantitative | Patient | Patients generally prefer IgRT treatment with fewer side effects | Preference for IEI treatments with fewer side effects |  |  |
| Gonzalez 2022 | Quantitative | Patient | Patients generally reported the most important treatment attributes of IgRT to be duration of side effects, followed by frequency of treatment, number of needles, setting of administration, infusion time, route of administration, and needing a nurse or not | Preference for IEI treatments with fewer side effects |  |  |
| Jones 2020b | Qualitative | Patient | “For a period of time, I did suffer really bad with shivers. When I’d finished my treatment, I would literally be like dithering, and I’d be sat in the car for a good 20 minutes/ half an hour, with the engine on and the heater blowing. I mentioned it to the nurses, and they were a bit concerned because they thought it could be an early stage of a reaction” | Preference for IEI treatments with fewer side effects |  |  |
| Jones 2020b | Qualitative | Patient | “My veins aren’t very good, and they had become scarred and it was getting harder and harder to get cannula’s in” | Preference for IEI treatments with fewer side effects |  |  |
| Jones 2020b | Qualitative | Patient | “Red spots, well not spots, but boils everywhere. Small boils everywhere. All my body I feel to scratch my body. And at the time, I was already out, so I ran back here. They gave me a Piriton, to sit down and relax and then it disappeared” | Preference for IEI treatments with fewer side effects |  |  |
| Jones 2020b | Qualitative | Patient | “You know to a new patient, they are going to refuse it, because people they don’t want a medicine that comes from blood, someone else. People are afraid… Yes, from someone else, you know like AIDS and things like that. So, you’re shocked you know, oh no, no, no, this situation, I don’t want it. But then you decide to take it because there are no other options” | Preference for IEI treatments with fewer side effects |  |  |
| Jones 2020a | Quantitative | Patient | A minority of patients always, often, or sometimes felt worried about having an unexpected reaction to their IgRT | Preference for IEI treatments with fewer side effects |  |  |
| Jones 2020a | Quantitative | Patient | A minority of patients always, often, or sometimes found that they get bruising at the site of the IgRT | Preference for IEI treatments with fewer side effects |  |  |
| Jones 2020a | Quantitative | Patient | Some patients always, often, or sometimes felt worried about catching another illness from their IgRT | Preference for IEI treatments with fewer side effects |  |  |
| Kutsa 2022 | Qualitative | Caregiver | So what we did was we made a kind of a pro/con chart, you know, we sat down and we wrote down, I wrote out all the different treatment options and, you know, what the positives and negatives are of each, which ones would need chemotherapy, which ones wouldn’t, and what possible side effects could happen and stuff like that. And that was a really, really important tool for us to help make the decisions. | Preference for IEI treatments with fewer side effects |  |  |
| Lechanska-Helman 2020 | Quantitative | Caregiver | A minority of caregivers preferred to treat their children subcutaneously vs intravenously as it had a smaller number of side effects | Preference for IEI treatments with fewer side effects |  |  |
| Smith 2021 | Qualitative | Caregiver | “His BMT was successful, however, he developed complications thought to be from cytoxin, liver engorged, ascites, which lead on to multisystem failure in ICU.” | Preference for IEI treatments with fewer side effects |  |  |
| Sultan 2017 | Qualitative | Patient | "I have fewer side effects" | Preference for IEI treatments with fewer side effects |  |  |
| Sultan 2017 | Qualitative | Patient | "I have more energy and fewer infections" | Preference for IEI treatments with fewer side effects |  |  |
| Sultan 2017 | Qualitative | Patient | "Sometimes, there is itching and swelling at the infusion sites" | Preference for IEI treatments with fewer side effects |  |  |
| Sultan 2017 | Qualitative | Patient | "There are bumps under my skin" | Preference for IEI treatments with fewer side effects |  |  |
| Sultan 2017 | Quantitative | Caregiver | Some caregivers report that limited side effects were a benefit of SCIg | Preference for IEI treatments with fewer side effects |  |  |
| Sultan 2017 | Quantitative | Patient | Some patients report that bumps under skin was a perceived issue of SCIg | Preference for IEI treatments with fewer side effects |  |  |
| Sultan 2017 | Quantitative | Patient | Some patients report that limited side effects were a benefit of SCIg | Preference for IEI treatments with fewer side effects |  |  |

**Supplementary Material 8.** Thematic synthesis for review theme of “Patients and caregivers place a high value on IEI treatments that maximize independence, efficiency, and flexibility.”

| **Study** | **Data Type** | **Perspective** | **Quote or Qualitized Narrative Summary** | **Code** | **Descriptive Theme** | **Overall Analytical Theme** |
| --- | --- | --- | --- | --- | --- | --- |
| AhmedMeelad 2022 | Qualitative | Caregiver | Every time that we had to go for IVIG, we were placed in daycare, because the IVIG procedure would take about 6h or so | Logistical burden of treatments | Preference for IEI treatments with minimal logistical and time burden | Patients and caregivers place a high value on IEI treatments that maximize independence, efficiency, and flexibility |
| AhmedMeelad 2022 | Qualitative | Caregiver | So his life now depends on the IVIG | Logistical burden of treatments |  |  |
| Cozon 2018 | Qualitative | Patient | I have moved eighty miles far from Lyon and on Friday evening after my infusion, I was feeling too tired to drive to home. | Logistical burden of treatments |  |  |
| Cozon 2018 | Qualitative | Patient | It is complicated when going out for holidays. I need to anticipate immunoglobulins supply. | Logistical burden of treatments |  |  |
| Cozon 2018 | Qualitative | Patient | It is more bothersome when I have to travel, planning material, storing the product in a cool place. | Logistical burden of treatments |  |  |
| Cozon 2018 | Qualitative | Patient | My employer did not appreciate that I had days off work every week. | Logistical burden of treatments |  |  |
| Franzese 2025 | Qualitative | Patient | “What was so time consuming was waiting for the pharmacy to bring the medicine, you know, for the IV, and sometimes I would wait two to three hours. But most of the time, I was at the hospital from 7:00 in the morning to maybe 6:00 or 7:00 that evening.” | Logistical burden of treatments |  |  |
| Franzese 2025 | Qualitative | Patient | “I was really under a lot of stress and the extra time driving there, even though it was only twenty or twenty-five minutes each way, it just added up and it took longer when you’re in a center waiting for nurses who are busy with other patients.” | Logistical burden of treatments |  |  |
| Franzese 2025 | Qualitative | Patient | “I live two miles from the medical center, so it was not an inconvenience to travel to the infusion clinic to get my medication. But my infusion could take up to four hours from start to finish once the product was infused into me. Half the battle would be the time it took to get called back from the waiting room to the infusion suite to get the nurse to have completed the vitals to all of that prep time. It made the process a good afternoon process from 12:00 to sometimes I would not get out till 5:30 or 6:00.” | Logistical burden of treatments |  |  |
| Franzese 2025 | Qualitative | Patient | “Having to go to a clinic for my infusions would be really hard. I live in a very rural area and would have to drive almost two hours each way. It would be hard on me to make such a long drive to receive my infusions and would be hard driving so far afterwards if I did not feel well. I take Benadryl as a pre-med and it makes me very tired. I do not have anyone to be able to commit time to drive me since they all have full time jobs. It works perfectly for me to be able to do them [the infusions] in the comfort of my home in the evenings when my husband is home. It would put a strain on us trying to find someone to drive me home afterwards [after in-clinic infusions].” | Logistical burden of treatments |  |  |
| Franzese 2025 | Qualitative | Patient | “The decision made me feel much more empowered as a patient, and it also gave me much more control over the process, the time of the infusions, and not having to go into a clinical setting to complete the infusions once the decision was made.” | Logistical burden of treatments |  |  |
| Franzese 2025 | Qualitative | Patient | “If I had to do this, go somewhere and do it rather than in the comfort of my own home, I know how much more complicated my life would be.” | Logistical burden of treatments |  |  |
| Franzese 2025 | Qualitative | Patient | “Being able to do this at home is so nice for me and I would not be able to juggle working full time and going to a clinic right now with my schedule. And so doing it at home is convenient and guarantees my access to the treatment. I would not have access to the treatment if I had to go to an infusion center.” | Logistical burden of treatments |  |  |
| Ito 2014 | Quantitative | Patient | Most patients like or like very much the ability to fit IGHy treatment into their schedules and its overall convenience, and a majority of patients like or like very much the administration frequency of IGHy | Logistical burden of treatments |  |  |
| Johansen 2026 | Qualitative | Patient | “To me it was a relief to do the treatment at home instead of having to go to the hospital every third week. I used an entire day, and at the time I worked full-time.” | Logistical burden of treatments |  |  |
| Jones 2020b | Qualitative | Patient | “You can’t infuse while you’re on your own. So, what we do is we just sort of work it round [husbands name] work timetable” | Logistical burden of treatments |  |  |
| Jones 2020b | Qualitative | Patient | “… it takes out a day and a half of your week, and it has an impact then on lots of things. If you think of your week, if you missed out on 36 hours of your week, I think you’d be, it’s hard to, something has to give doesn’t it, in that way. Sometimes I can get a bit, a bit miffed about it” | Logistical burden of treatments |  |  |
| Jones 2020b | Qualitative | Patient | “And I’ve got a daughter who’s 17 now. I mean she was a lot younger then and when you’re in hospital, you’re thinking how they are at home. You know, X [husband] was working all the time and X [daughter] had to go between different grandparents and things” “P: Because it were depressing me that much, you know I need to come all time so I just kept, and I were working as well. I had to keep having days off work and stuff like that, and it was getting me right down like. So I just thought ‘stuff it’, I weren’t bothered, and I probably went a few months, I’ve done it a few times you know. I: Stop taking it? P: It’s got me down that much like, and because when I start getting like that, that has an effect at home, you know what I mean with kids and every- thing and our lass. So I just stopped going. But then I started to like feel drained in myself and everything and get more chest infections and stuff. Anyway, I’ve always ended up coming back and that so” | Logistical burden of treatments |  |  |
| Jones 2020b | Qualitative | Patient | “Because it was depressing me that much, you know I need to come all time, so I just kept, and I were working as well. I had to keep having days off work and stuff like that, and it was getting me right down like. So, I just thought ‘stuff it’, I weren’t bothered, and I probably went a few months, I’ve done it a few times you know” | Logistical burden of treatments |  |  |
| Jones 2020b | Qualitative | Patient | “Because you can’t put your bottles in the same fridge as a normal fridge, as your food. So, I have a separate fridge, so that’s very good | Logistical burden of treatments |  |  |
| Jones 2020b | Qualitative | Patient | “I childmind for my youngest daughter Monday, Tuesday, Wednesday evenings only for an hour. And it’s not the treatment’s fault. If transport is late, I don’t get home on time to go and childmind. So now they say, ‘oh is it your treatment week mum?’ And I say, ‘yes it’s my treatment week” | Logistical burden of treatments |  |  |
| Jones 2020b | Qualitative | Patient | “I phoned them and said ‘look, I’ve been driving around for 20 minutes, and I cannot find parking anywhere’. I said ‘I’ve tried the road, I’ve tried in- side. So if I don’t actually turn up to this appointment, you know it’s not lack of trying” | Logistical burden of treatments |  |  |
| Jones 2020b | Qualitative | Patient | “I suppose on the back of that, the other impact it had is things like social life and feeling normal alongside your friends, because I’d go into hospital once a week, once a fortnight, and probably spend 2 or 3 days recovering from that, just getting to feel well enough again” | Logistical burden of treatments |  |  |
| Jones 2020b | Qualitative | Patient | “I think it’s just because I’ve been doing it for like 32/33 years, and that’s quite a big commitment isn’t it, so that for me at times, I just get every three weeks thinking ‘argh here we go again’, and I really wish I could have like a break, and not have to do it this time, but I know I can’t, so I do.” | Logistical burden of treatments |  |  |
| Jones 2020b | Qualitative | Patient | “If you went away for three weeks, you’d be ok, but I think if it was any longer than that, so I don’t think I can go on a round the world cruise without making some special arrangements” | Logistical burden of treatments |  |  |
| Jones 2020b | Qualitative | Patient | “It’s easier if you know you’re coming to hospital, you know you’re going to have half a day. You just turn up, you sit, you’re done, you go home. You don’t have to worry about it. Whereas at home you’re obviously, you’re in your home environment, various things come up. You’ve obviously got to try and keep it consistent, you know, infusion day, you know make sure obviously your products out the fridge, make sure all your supplies are there, it’s just more to think about. For me, this is easier” | Logistical burden of treatments |  |  |
| Jones 2020b | Qualitative | Patient | “Now, I’m catching like three buses to get here and it’s taking a lot longer” | Logistical burden of treatments |  |  |
| Jones 2020b | Qualitative | Patient | “So I prepare- I’ve got this nice little table that I use, and it’s easy to sterile and everything. I get my delivery every three months. I make packs up. So everything I need for that week, so I make me 12 packs up ready.” | Logistical burden of treatments |  |  |
| Jones 2020b | Qualitative | Patient | “We had to be up for 6 o’ clock this morning. Well, ready for 6am” | Logistical burden of treatments |  |  |
| Jones 2020b | Qualitative | Patient | “When it was coming around to treatment, it did used to depress me like because I know that when I get it I’m going to be here for like 5 hours or something like that, and it’s tiring, sat in hospitals” | Logistical burden of treatments |  |  |
| Jones 2020b | Qualitative | Patient | “You think ‘I’ve got to put a whole day aside just for this…it’s 25 miles | Logistical burden of treatments |  |  |
| Jones 2020a | Quantitative | Patient | A minority of patients always, often, or sometimes found it labour intensive to set up the equipment needed for their IgRT (e.g., pumps) | Logistical burden of treatments |  |  |
| Jones 2020a | Quantitative | Patient | A minority of patients always, often, or sometimes found that planning ahead to have their IgRT takes effort | Logistical burden of treatments |  |  |
| Lechanska-Helman 2020 | Quantitative | Caregiver | A minority of caregivers preferred to treat their children subcutaneously vs intravenously as infusions did not interfere with their work, their child’s preschool, and other activities | Logistical burden of treatments |  |  |
| Margolis 2017 | Qualitative | Patient | "I forget on purpose because I don’t want to take them. I’ve been taking medication so long that it’s irritating, annoying and I don’t want to take it. Everything should be fun at my age and I shouldn’t have to take 12 pills [a day].” | Logistical burden of treatments |  |  |
| Ponsford 2015 | Qualitative | Patient | Annoying doing every week although a great treatment. More painful, would last 24 h. Reminded me I was ill. More inconvenient, I had to take it with me when I travelled | Logistical burden of treatments |  |  |
| Ponsford 2015 | Qualitative | Patient | New starter. Planning to travel therefore keen for least invasive replacement regimen | Logistical burden of treatments |  |  |
| Ponsford 2015 | Qualitative | Patient | Really happy with this. Only painful for a few hours. Only infuse every 3 weeks now, so barely remember I have to do it. No need to take it with me when I travel | Logistical burden of treatments |  |  |
| Raspa 2020 | Qualitative | Caregiver | “They were doing bloodwork almost every week. You’re waiting for changes but there weren’t changes; the levels were still the same. It was hard. It was just a waiting game. Waiting to see when I would be out of the hospital or how long I would be there.” | Logistical burden of treatments |  |  |
| Sultan 2017 | Qualitative | Patient | "It can be difficult to fit the treatment into my schedule (school, holidays)" | Logistical burden of treatments |  |  |
| Sultan 2017 | Quantitative | Patient | A majority of patients report that convenience was a benefit of SCIg | Logistical burden of treatments |  |  |
| Sultan 2017 | Quantitative | Caregiver | A minority of caregivers report that convenience was a benefit of SCIg | Logistical burden of treatments |  |  |
| Sultan 2017 | Qualitative | Patient | It takes time to prepare and to administer the treatment | Logistical burden of treatments |  |  |
| Sultan 2017 | Quantitative | Patient | Some patients report that needing planning was a perceived issue of SCIg | Logistical burden of treatments |  |  |
| AhmedMeelad 2022 | Qualitative | Caregiver | Every time that we had to go for IVIG, we were placed in daycare, because the IVIG procedure would take about 6h or so | Preference for less frequent and shorter duration IEI treatments |  |  |
| Eltan 2022 | Quantitative | Patient | A majority of patients reported that having a shorter infusion duration was a reason for being satisfied with SCIg | Preference for less frequent and shorter duration IEI treatments |  |  |
| Espanol 2012 | Quantitative | Patient/Caregiver | A minority of patients and caregivers reported treatment with quicker administration as an ideal treatment | Preference for less frequent and shorter duration IEI treatments |  |  |
| Espanol 2012 | Quantitative | Patient/Caregiver | Some patients and caregivers reported treatment with longer times between infusions as an ideal treatment | Preference for less frequent and shorter duration IEI treatments |  |  |
| Espanol 2014 | Quantitative | Patient/Caregiver | Patients and caregivers preferred a shorter treatment duration during IgRT | Preference for less frequent and shorter duration IEI treatments |  |  |
| Espanol 2014 | Quantitative | Patient/Caregiver | Patients and caregivers preferred monthly administration of IgRT when compared with either fort nightly or weekly administration | Preference for less frequent and shorter duration IEI treatments |  |  |
| Gardulf 1995 | Qualitative | Patient | It is time-consuming, I want two syringe drivers at home as well | Preference for less frequent and shorter duration IEI treatments |  |  |
| Gardulf 1995 | Qualitative | Patient | The negative thing is that you have to take the gammaglobulin regularly | Preference for less frequent and shorter duration IEI treatments |  |  |
| Gonzalez 2022 | Quantitative | Patient | Patients generally prefer IgRT treatment with less frequent administration intervals | Preference for less frequent and shorter duration IEI treatments |  |  |
| Gonzalez 2022 | Quantitative | Patient | Patients generally prefer IgRT treatment with shorter infusion times | Preference for less frequent and shorter duration IEI treatments |  |  |
| Ito 2014 | Quantitative | Patient | Most patients like or like very much the ability to fit IGHy treatment into their schedules and its overall convenience, and a majority of patients like or like very much the administration frequency of IGHy | Preference for less frequent and shorter duration IEI treatments |  |  |
| Jones 2020b | Qualitative | Patient | “I think it’s just because I’ve been doing it for like 32/33 years, and that’s quite a big commitment isn’t it, so that for me at times, I just get every three weeks thinking ‘argh here we go again’, and I really wish I could have like a break, and not have to do it this time, but I know I can’t, so I do.” | Preference for less frequent and shorter duration IEI treatments |  |  |
| Jones 2020b | Qualitative | Patient | “When it was coming around to treatment, it did used to depress me like because I know that when I get it I’m going to be here for like 5 hours or something like that, and it’s tiring, sat in hospitals” | Preference for less frequent and shorter duration IEI treatments |  |  |
| Jones 2020a | Quantitative | Patient | A minority of patients always, often, or sometimes felt that their IgRT is too frequent | Preference for less frequent and shorter duration IEI treatments |  |  |
| Jones 2020a | Quantitative | Patient | A minority of patients always, often, or sometimes felt that their IgRT takes up too much time | Preference for less frequent and shorter duration IEI treatments |  |  |
| Jones 2020a | Quantitative | Patient | A minority of patients always, often, or sometimes found that they worry about the time needed to have their IgRT | Preference for less frequent and shorter duration IEI treatments |  |  |
| Lechanska-Helman 2020 | Quantitative | Caregiver | A majority of caregivers preferred to treat their children intravenously vs subcutaneously as it made it possible for the children to receive treatment once per month | Preference for less frequent and shorter duration IEI treatments |  |  |
| Mohamed 2012 | Quantitative | Patient/Caregiver | Patients and caregivers generally prefer IgRT that have less frequent treatment intervals | Preference for less frequent and shorter duration IEI treatments |  |  |
| Mohamed 2012 | Quantitative | Patient/Caregiver | Patients and caregivers generally prefer IgRT with shorter infusion durations | Preference for less frequent and shorter duration IEI treatments |  |  |
| Ponsford 2015 | Qualitative | Patient | Able to infuse much quicker: feeling much happier. Some wear off with fSCIg noted | Preference for less frequent and shorter duration IEI treatments |  |  |
| Ponsford 2015 | Qualitative | Patient | Annoying doing every week although a great treatment. More painful, would last 24 h. Reminded me I was ill. More inconvenient, I had to take it with me when I travelled | Preference for less frequent and shorter duration IEI treatments |  |  |
| Ponsford 2015 | Qualitative | Patient | Better infusing 3-weekly but changing bottles over can be fiddly | Preference for less frequent and shorter duration IEI treatments |  |  |
| Ponsford 2015 | Qualitative | Patient | Busy lifestyle, preferred fewer infusions | Preference for less frequent and shorter duration IEI treatments |  |  |
| Ponsford 2015 | Qualitative | Patient | Difficulty self-cannulating, gravity infusion slow, felt very lethargic. Keen to avoid weekly regimen but still remain at home | Preference for less frequent and shorter duration IEI treatments |  |  |
| Ponsford 2015 | Qualitative | Patient | Greater time between treatments a big benefit | Preference for less frequent and shorter duration IEI treatments |  |  |
| Ponsford 2015 | Qualitative | Patient | Lifestyle preference – keen to reduce infusion frequency of weekly SCIg | Preference for less frequent and shorter duration IEI treatments |  |  |
| Ponsford 2015 | Qualitative | Patient | New epilepsy diagnosis – unable to drive to day unit. Weekly SCIg infusions an increasing strain – keen for alternative | Preference for less frequent and shorter duration IEI treatments |  |  |
| Ponsford 2015 | Qualitative | Patient | Rate-related side effects with IVIg – felt tired and drained. Infusions slow, took all day | Preference for less frequent and shorter duration IEI treatments |  |  |
| Ponsford 2015 | Qualitative | Patient | Really happy with this. Only painful for a few hours. Only infuse every 3 weeks now, so barely remember I have to do it. No need to take it with me when I travel | Preference for less frequent and shorter duration IEI treatments |  |  |
| Ponsford 2015 | Qualitative | Patient | Still effective, more convenient being 3-weekly | Preference for less frequent and shorter duration IEI treatments |  |  |
| Ponsford 2015 | Qualitative | Patient | Suffered rate-related side effects with previous home IVIg. Preferred fewer infusions, due to busy lifestyle | Preference for less frequent and shorter duration IEI treatments |  |  |
| Ponsford 2015 | Qualitative | Patient | Wanted to do infusion at home but not weekly. Large dose requirement with wear-off on 3-weekly IVIg | Preference for less frequent and shorter duration IEI treatments |  |  |
| Reid 2014 | Quantitative | Patient/Caregiver | Patients and caregivers generally ranked lost time as the most unpleasant experience related to IVIG, followed by the IV infusion process, the waiting time, travel, adverse events, and the registration process | Preference for less frequent and shorter duration IEI treatments |  |  |
| Runken 2017 | Quantitative | Patient | A majority of patients agree that they would prefer one longer infusion over several short infusions each month | Preference for less frequent and shorter duration IEI treatments |  |  |
| Rutland 2025 | Quantitative | Caregiver | Patients generally place a high importance on the speed of IgRT pump setup in the decision-making process for choosing a particular pump for therapy | Preference for less frequent and shorter duration IEI treatments |  |  |
| Rutland 2025 | Quantitative | Caregiver | Patients generally consider fast and easy setup, compact and portable design, and minimal steps involved in setting up the IgRT pump as important in the decision-making process for choosing a mechanical pump for therapy | Preference for less frequent and shorter duration IEI treatments |  |  |
| Sultan 2017 | Qualitative | Patient | "I have to do it every week" | Preference for less frequent and shorter duration IEI treatments |  |  |
| Sultan 2017 | Qualitative | Patient | "SCIg takes less time. IVIg took one day" | Preference for less frequent and shorter duration IEI treatments |  |  |
| Sultan 2017 | Quantitative | Patient | A minority of patients report that frequency of administration was a perceived issue of SCIg | Preference for less frequent and shorter duration IEI treatments |  |  |
| Sultan 2017 | Quantitative | Patient | A minority of patients report that length of administration was a perceived issue of SCIg | Preference for less frequent and shorter duration IEI treatments |  |  |
| Sultan 2017 | Quantitative | Caregiver | Some caregivers report that that frequency of administration was a perceived issue of SCIg | Preference for less frequent and shorter duration IEI treatments |  |  |
| Sultan 2017 | Quantitative | Caregiver | Some caregivers report that that length of administration was a perceived issue of SCIg | Preference for less frequent and shorter duration IEI treatments |  |  |
| Chatelier 2014 | Quantitative | Patient | Most patients are interested in home-based IgRT | Preference for home based IgRT | Preference for autonomy in IgRT administration |  |
| Cozon 2018 | Qualitative | Patient | I no more had to drive to the hospital which took me 3 hours. | Preference for home based IgRT |  |  |
| Cozon 2018 | Qualitative | Patient | I could do infusions on Saturday morning. | Preference for home based IgRT |  |  |
| Eltan 2022 | Quantitative | Patient | A majority of patients reported that being able to self-administer at home was a reason for being satisfied with SCIg | Preference for home based IgRT |  |  |
| Espanol 2014 | Quantitative | Patient/Caregiver | Patients and caregivers preferred administration of IgRT at home rather than in a doctor’s office, clinic, or hospital | Preference for home based IgRT |  |  |
| Franzese 2025 | Qualitative | Patient | “What was so time consuming was waiting for the pharmacy to bring the medicine, you know, for the IV, and sometimes I would wait two to three hours. But most of the time, I was at the hospital from 7:00 in the morning to maybe 6:00 or 7:00 that evening.” | Preference for home based IgRT |  |  |
| Franzese 2025 | Qualitative | Patient | “I was really under a lot of stress and the extra time driving there, even though it was only twenty or twenty-five minutes each way, it just added up and it took longer when you’re in a center waiting for nurses who are busy with other patients.” | Preference for home based IgRT |  |  |
| Franzese 2025 | Qualitative | Patient | “I live two miles from the medical center, so it was not an inconvenience to travel to the infusion clinic to get my medication. But my infusion could take up to four hours from start to finish once the product was infused into me. Half the battle would be the time it took to get called back from the waiting room to the infusion suite to get the nurse to have completed the vitals to all of that prep time. It made the process a good afternoon process from 12:00 to sometimes I would not get out till 5:30 or 6:00.” | Preference for home based IgRT |  |  |
| Franzese 2025 | Qualitative | Patient | “Having to go to a clinic for my infusions would be really hard. I live in a very rural area and would have to drive almost two hours each way. It would be hard on me to make such a long drive to receive my infusions and would be hard driving so far afterwards if I did not feel well. I take Benadryl as a pre-med and it makes me very tired. I do not have anyone to be able to commit time to drive me since they all have full time jobs. It works perfectly for me to be able to do them [the infusions] in the comfort of my home in the evenings when my husband is home. It would put a strain on us trying to find someone to drive me home afterwards [after in-clinic infusions].” | Preference for home based IgRT |  |  |
| Franzese 2025 | Qualitative | Patient | “The decision made me feel much more empowered as a patient, and it also gave me much more control over the process, the time of the infusions, and not having to go into a clinical setting to complete the infusions once the decision was made.” | Preference for home based IgRT |  |  |
| Franzese 2025 | Qualitative | Patient | “If I had to do this, go somewhere and do it rather than in the comfort of my own home, I know how much more complicated my life would be.” | Preference for home based IgRT |  |  |
| Franzese 2025 | Qualitative | Patient | “Being able to do this at home is so nice for me and I would not be able to juggle working full time and going to a clinic right now with my schedule. And so doing it at home is convenient and guarantees my access to the treatment. I would not have access to the treatment if I had to go to an infusion center.” | Preference for home based IgRT |  |  |
| Gardulf 1995 | Qualitative | Patient | Home treatment gives you the opportunity to live like a 'normal' human being. If anyone feels disabled by the antibody deficiency, home treatment reduces this feeling to a minimum | Preference for home based IgRT |  |  |
| Gardulf 1995 | Qualitative | Patient | I can travel without any problems Now I feel that I am contributing to my own well-being, I am no longer just a 'patient' or a 'case' | Preference for home based IgRT |  |  |
| Gardulf 1995 | Qualitative | Patient | The home treatment means greater independence and flexibility | Preference for home based IgRT |  |  |
| Gardulf 2004 | Quantitative | Patient | All patients previously on SCIG and a majority of patients previously on IVIG prefer home-based SCIG | Preference for home based IgRT |  |  |
| Gonzalez 2022 | Quantitative | Patient | Patients generally prefer IgRT treatment at home compared to at clinic | Preference for home based IgRT |  |  |
| Johansen 2026 | Qualitative | Patient | “To me it was a relief to do the treatment at home instead of having to go to the hospital every third week. I used an entire day, and at the time I worked full-time.” | Preference for home based IgRT |  |  |
| Johansen 2026 | Qualitative | Patient | “I could not bear being in the department together with the seriously ill cancer patients (…) I was younger and well and felt stigmatised when getting treatment at the hospital.” | Preference for home based IgRT |  |  |
| Johansen 2026 | Qualitative | Patient | “You avoid going to the hospital and being reminded of being ill.” | Preference for home based IgRT |  |  |
| Jones 2020b | Qualitative | Patient | “It’s took a lot less time, not having to go to hospital. You haven’t got to waste a day, or waste half a day in the hospital, wasting doctor and nurses time... but now I just go to work, come home one day, have my tea, and then start this treatment straight afterwards.” | Preference for home based IgRT |  |  |
| Meckley 2020 | Quantitative | Patient | Most patients prefer to administer their IgRT at home | Preference for home based IgRT |  |  |
| Mohamed 2012 | Quantitative | Patient/Caregiver | Patients and caregivers generally prefer administering IgRT at home rather than at a doctor's office, hospital, or clinic | Preference for home based IgRT |  |  |
| Myers 2025 | Quantitative | Patient | Most patients prefer home infusion over hospital/infusion settings due to reduced exposure risk | Preference for home based IgRT |  |  |
| Ponsford 2015 | Qualitative | Patient | Difficulty self-cannulating, gravity infusion slow, felt very lethargic. Keen to avoid weekly regimen but still remain at home | Preference for home based IgRT |  |  |
| Ponsford 2015 | Qualitative | Patient | Wanted to do infusion at home but not weekly. Large dose requirement with wear-off on 3-weekly IVIg | Preference for home based IgRT |  |  |
| Sultan 2017 | Qualitative | Patient | "It’s easier to do the treatment at home and I don’t have to go to the hospital every month" | Preference for home based IgRT |  |  |
| Sultan 2017 | Quantitative | Patient | A majority of patients report that being able to administer at home was a benefit of SCIg | Preference for home based IgRT |  |  |
| Sultan 2017 | Quantitative | Caregiver | A minority of caregivers report that being able to administer at home was a benefit of SCIg | Preference for home based IgRT |  |  |
| Eltan 2022 | Quantitative | Patient | A majority of patients reported that being able to self-administer at home was a reason for being satisfied with SCIg | Preference for the ability to self-administer IgRT |  |  |
| Espanol 2014 | Quantitative | Patient/Caregiver | Patients and caregivers preferred self-administration of IgRT compared to administration by a health care professional | Preference for the ability to self-administer IgRT |  |  |
| Gonzalez 2022 | Quantitative | Patient | Patients generally prefer IgRT treatment without a nurse than with a nurse | Preference for the ability to self-administer IgRT |  |  |
| Meckley 2020 | Quantitative | Patient | Almost all patients liked or liked very much the ability to administer their IgRT without supervision | Preference for the ability to self-administer IgRT |  |  |
| Meckley 2020 | Quantitative | Patient | Almost all patients liked or liked very much the potential to self-administer their IgRT | Preference for the ability to self-administer IgRT |  |  |
| Mohamed 2012 | Quantitative | Patient/Caregiver | Patients and caregivers generally prefer to self-administer IgRT rather than having an appointment with a HCP | Preference for the ability to self-administer IgRT |  |  |
| Gardulf 1995 | Qualitative | Patient | I can travel without any problems Now I feel that I am contributing to my own well-being, I am no longer just a 'patient' or a 'case' | Preference for IgRT with greater autonomy |  |  |
| Meckley 2020 | Quantitative | Patient | Almost all patients liked or liked very much being able to fit their IgRT into their own schedule | Preference for IgRT with greater autonomy |  |  |
| NapiorkowskaBaran 2021 | Quantitative | Patient | A majority of patients report interest in having home delivery of drugs continuing after the COVID-19 pandemic | Preference for IgRT with greater autonomy |  |  |
| Reid 2014 | Quantitative | Patient/Caregiver | Needing a nurse for home based IVIG was related to more unwillingness to swap from hospital to home based IVIG | Preference for IgRT with greater autonomy |  |  |
| Sultan 2017 | Qualitative | Patient | "I can choose when I want to do the treatment" | Preference for IgRT with greater autonomy |  |  |
| Sultan 2017 | Qualitative | Patient | "I don’t have to miss school because I do the treatment at home" | Preference for IgRT with greater autonomy |  |  |
| Sultan 2017 | Quantitative | Caregiver | Some caregivers report that more autonomy was a benefit of SCIg | Preference for IgRT with greater autonomy |  |  |
| Sultan 2017 | Quantitative | Patient | Some patients report that more autonomy was a benefit of SCIg | Preference for IgRT with greater autonomy |  |  |

**Supplementary Material 9.** Thematic synthesis for review theme of “Patients and caregivers place a high value on effective transitions from pediatric to adult care services.”

| **Study** | **Data Type** | **Perspective** | **Quote or Qualitized Narrative Summary** | **Code** | **Descriptive Theme** | **Overall Analytical Theme** |
| --- | --- | --- | --- | --- | --- | --- |
| Agnihotri 2021 | Quantitative | Patient/Caregiver | A majority of caregivers and most patients believe a written individualized plan for transition should be provided | Burden of transition from pediatric to adult care | Burden of transition from pediatric to adult care | Patients and caregivers place a high value on effective transitions from pediatric to adult care services |
| Agnihotri 2021 | Quantitative | Patient/Caregiver | A majority of caregivers and patients believe that providers should prepare IEI patients for transition around ages 15-17 | Burden of transition from pediatric to adult care |  |  |
| Agnihotri 2021 | Quantitative | Patient/Caregiver | All caregivers and most patients agree that during transition patients should be educated about medications, patient condition, and symptoms that require seeking health care | Burden of transition from pediatric to adult care |  |  |
| Agnihotri 2021 | Quantitative | Patient/Caregiver | Most caregivers and almost all patients prefer a transition coordinator for pediatric to adult care | Burden of transition from pediatric to adult care |  |  |
| Margolis 2017 | Qualitative | Patient | "There was no transition. It just kind of happened.” | Burden of transition from pediatric to adult care |  |  |
| Margolis 2017 | Qualitative | Patient | “[The doctor] pesters me and she’s persistent and I don’t want to disappoint her. I finally got myself organized. The doctors motivated me in the past few years and I started taking care of things myself.” | Burden of transition from pediatric to adult care |  |  |
| Margolis 2017 | Qualitative | Patient | “A pamphlet outlining what goes into a visit starting with travel, reservations, clinic appointment.” | Burden of transition from pediatric to adult care |  |  |
| Margolis 2017 | Qualitative | Patient | “Do more walk-throughs on the unit to get familiar with the unit and nurses.” | Burden of transition from pediatric to adult care |  |  |
| Margolis 2017 | Qualitative | Patient | “Encourage patients to be an active participant. It’s important to know what and why.” | Burden of transition from pediatric to adult care |  |  |
| Margolis 2017 | Qualitative | Patient | “Have a support system with other patients. Talking to other patients about transition would help. I would be willing to talk to others.” | Burden of transition from pediatric to adult care |  |  |
| Margolis 2017 | Qualitative | Patient | “Have the doctor tell parents to talk to their kids at age 15 or 16.” | Burden of transition from pediatric to adult care |  |  |
| Margolis 2017 | Qualitative | Patient | “Make sure the patient is comfortable in the setting they are in. They need to (1) understand the importance of taking medicine, (2) if getting sick contact the doctor right away. (3) Understanding the disease is highly important and (4) Know it is possible to live a full healthy lifestyle if staying on top of what they need to know.” | Burden of transition from pediatric to adult care |  |  |
| Margolis 2017 | Qualitative | Patient | “My parents always encouraged me to ask questions. The younger the better. My mom got scolded by the doctors for not taking a more active role but she said she wasn’t always going to be around.” | Burden of transition from pediatric to adult care |  |  |
| Margolis 2017 | Qualitative | Patient | “Sit down one on one with your doctor to talk about your meds and disease.” | Burden of transition from pediatric to adult care |  |  |
| Margolis 2017 | Qualitative | Patient | “Start young, like around 15 or 16, a few years before. Talk about their flights and appointments with their parents there but not doing it for them.” | Burden of transition from pediatric to adult care |  |  |
| Margolis 2017 | Qualitative | Patient | “You gotta work with your parents to learn your disease and medications while you’re growing up.” | Burden of transition from pediatric to adult care |  |  |
| Ouimet 2023 | Qualitative | Patient | "Yeah, you know I don’t feel super secure because they forget. Not that they forget, it’s normal to forget, but you know, that they don’t read everything.” | Burden of transition from pediatric to adult care |  |  |
| Ouimet 2023 | Qualitative | Caregiver | “I was panicking about who is going to follow her and how it’s going to work.” | Burden of transition from pediatric to adult care |  |  |
| Ouimet 2023 | Qualitative | Caregiver | “I’m just telling you that we would have felt … Less nervous. Because when they tell us: “You’re going to meet [adult immunologist].” But who is he? Are we going to feel comfortable? Will we like him? Will we feel understood? We’ve been in the void for eighteen years.” | Burden of transition from pediatric to adult care |  |  |
| Ouimet 2023 | Qualitative | Caregiver | “It’s hard and it’s a lot of stress for the youth with the medication. We had a family doctor who took charge of her, so it was the family doctor who did the renewals of specialists’ medication, but that’s not ideal either. So, it’s a lot of questions about who is going to do the follow-up, how. It’s a lot of unknowns too.” | Burden of transition from pediatric to adult care |  |  |
| Ouimet 2023 | Qualitative | Caregiver | “It’s like him that was doing the briefing of his own case, without them reading the file, because his file is so thick, maybe I understand those who don’t read. It’s so big. There were some who sympathized with him, who understood him very well. Since then, they try to understand, to help him, to the best of their ability. On the other hand, there were some who were really insensitive, as if they were dealing with a number, not a person, not a human being. It’s just like a number in the hospital, that’s all.” | Burden of transition from pediatric to adult care |  |  |
| Ouimet 2023 | Qualitative | Caregiver | “Once in adult care, he was considered a full-fledged adult, whereas he could not be called that, a young adult, not like a real adult. They imposed certain things on him that he was against, he was a teenager, you shouldn’t rush a teenager.” | Burden of transition from pediatric to adult care |  |  |
| Ouimet 2023 | Qualitative | Caregiver | “to know to whom you’re going, and not be on waiting lists waiting until they call you. Like meeting [the adult immunologist] at [pediatric hospital], I think, that was reassuring for [my youth].” | Burden of transition from pediatric to adult care |  |  |
| Ouimet 2023 | Qualitative | Caregiver | Some participants did not know that the transition occurred at 18 years old and thought that “in certain cases [the pediatric] hospital kept the child until 21 years old” (CG-6). As this caregiver further explained: “It’s weird, we knew 18 was the adult age, but we thought since our daughter has a mental age of seven, eight or whatever, that they would keep her for her exceptional conditions. But no.” | Burden of transition from pediatric to adult care |  |  |
| Agnihotri 2021 | Quantitative | Patient/Caregiver | A majority of caregivers and patients believe that providers should prepare IEI patients for transition around ages 15-17 | Desire for more knowledge and resources regarding IEI | Preference for care and counseling from HCPs knowledgeable about IEI diagnosis, management, and resources |  |
| Agnihotri 2021 | Quantitative | Patient/Caregiver | All caregivers and most patients agree that during transition patients should be educated about medications, patient condition, and symptoms that require seeking health care | Desire for more knowledge and resources regarding IEI |  |  |
| Agnihotri 2021 | Quantitative | Patient/Caregiver | Most caregivers and almost all patients prefer a transition coordinator for pediatric to adult care | Desire for more knowledge and resources regarding IEI |  |  |
| AhmadAzahari 2023 | Qualitative | Caregiver | …public awareness on genetic testing is very low, moreover, for Malay community they believe in superstition saying that we did something wrong. | Desire for more knowledge and resources regarding IEI |  |  |
| AhmadAzahari 2023 | Qualitative | Caregiver | …when I first heard about genetic testing, I also don’t know what it is actually. | Desire for more knowledge and resources regarding IEI |  |  |
| AhmadAzahari 2023 | Qualitative | Caregiver | …but I find it difficult to understand (genetic test and PID), so I keep asking the doctors and they say that if I want more information, I should also try Googling it. | Desire for more knowledge and resources regarding IEI |  |  |
| AhmadAzahari 2023 | Qualitative | Caregiver | …I am not aware about the cost and was not informed about it either (cost for genetic testing). | Desire for more knowledge and resources regarding IEI |  |  |
| AhmadAzahari 2023 | Qualitative | Caregiver | …I prefer both ways, whether it’s obtaining information online or through doctors. | Desire for more knowledge and resources regarding IEI |  |  |
| AhmadAzahari 2023 | Qualitative | Caregiver | …Initially, we relied solely on the doctors for information, but at that time, we didn’t have internet access. So, we turned to our friends who had access to Google and printed out the information (genetic disease) for us. | Desire for more knowledge and resources regarding IEI |  |  |
| AhmedMeelad 2022 | Qualitative | Caregiver | Before that I haven't event met anyone from the immunologist team, they just gave me the name of the disease, the category. So then I looked it up. Then I got more confused than before, and I started to think of getting on ways of how to get him out from the hospital. | Desire for more knowledge and resources regarding IEI |  |  |
| AhmedMeelad 2022 | Qualitative | Caregiver | But if we have the kind of society that lacks awareness and knowledge or we are surrounded with those kinds of people that does not have the awareness about this disease. | Desire for more knowledge and resources regarding IEI |  |  |
| AhmedMeelad 2022 | Qualitative | Caregiver | Previously I only knew the type like XXX. When I entered MyPOPI, it was then I got to know that there are other types of PID that I haven't even heard about | Desire for more knowledge and resources regarding IEI |  |  |
| Allen 2022 | Quantitative | Patient | A majority of patients are interested in receiving more information on adjusting to life post-treatment | Desire for more knowledge and resources regarding IEI |  |  |
| Allen 2022 | Quantitative | Patient | A majority of patients are interested in receiving more information on returning to school/work | Desire for more knowledge and resources regarding IEI |  |  |
| Allen 2022 | Quantitative | Patient | A minority of patients are interested in receiving more information nutrition and healthy eating | Desire for more knowledge and resources regarding IEI |  |  |
| Allen 2022 | Quantitative | Patient | A minority of patients are interested in receiving more information on adjusting to diagnosis and treatment | Desire for more knowledge and resources regarding IEI |  |  |
| Beers 2025 | Qualitative | Patient | “I’ve also learned a lot of the medical terminology for my issues; that makes me feel a little bit more in control whenever I’m talking to a doctor.” | Desire for more knowledge and resources regarding IEI |  |  |
| Beers 2025 | Qualitative | Patient | “If you know more about your illness, you can live your life better. … If you don't know much about your illness, you keep struggling and keep asking, ‘Why is this happening this to me? What is happening? Why do I get all those infections?’” | Desire for more knowledge and resources regarding IEI |  |  |
| Clark 2025 | Qualitative | Caregiver | “A sourcing of information about your condition that is correct and just look here for what you need, that would be helpful…and we did get that to an extent, but something as simple as what’s the life expectancy? I don’t think we got that. I was finding that out from medical journal… but because you have these kinds of questions, which then you spiral…and you just end up Googling…” | Desire for more knowledge and resources regarding IEI |  |  |
| Clark 2025 | Qualitative | Caregiver | “I think the hardest thing is the uncertainty with the whole thing. There are no answers, and you have no idea of the outcome.” | Desire for more knowledge and resources regarding IEI |  |  |
| Clark 2025 | Qualitative | Caregiver | “We’re really happy with really with our doctors, but they don’t necessarily explain things to the kids well… no one’s really ever explained to him what’s going on.” | Desire for more knowledge and resources regarding IEI |  |  |
| Davidson 2024 | Qualitative | Patient | [E]very time we’d go to adoctor, it would be “Well, do you think this is related?” and you’d hear ‘Oh, well, maybe.’ ‘Oh, I don’t think so,’ and then you’d get other doctors who are like ‘Yeah, I’m 100% sure it’s related,’ and now, they’re pretty well sure it’s related. So, when we got this [PIK3CD GOF disorder] diagnosis … It started terrifying me because none of [my brother’s] symptoms were the same as my symptoms. So, I was wonder[ing] if it was related to the mutation and it made me fear for my kids. | Desire for more knowledge and resources regarding IEI |  |  |
| Gardulf 1995 | Quantitative | Patient | Patients generally desired for more knowledge about their antibody deficiency | Desire for more knowledge and resources regarding IEI |  |  |
| Jones 2020b | Qualitative | Patient | “I thought it was a quick-fix thing. Obviously, what I didn’t hear, which I’ve now got the grip of, it has been explained to me, that of course, it isn’t is it? It’s long-term, well it’s for life basically” | Desire for more knowledge and resources regarding IEI |  |  |
| Joseph 2016 | Qualitative | Caregiver | “I think personally, as a newborn parent, I had no idea of a lot of things and I think it’s great that it’s mandatory because you’re not aware of diseases and things that babies are diagnosed with. …we all have kids with medical issues, but I think that before I had my children [if] they had asked me, ‘Do you want [newborn screening]?’ No, I wouldn't want them to be tested. Now I would answer, absolutely ‘Yeah, test’.” | Desire for more knowledge and resources regarding IEI |  |  |
| Kutsa 2022 | Qualitative | Caregiver | So what we did was we made a kind of a pro/con chart, you know, we sat down and we wrote down, I wrote out all the different treatment options and, you know, what the positives and negatives are of each, which ones would need chemotherapy, which ones wouldn’t, and what possible side effects could happen and stuff like that. And that was a really, really important tool for us to help make the decisions. | Desire for more knowledge and resources regarding IEI |  |  |
| Kutsa 2022 | Qualitative | Caregiver | We just had to have faith in his doctors and ask a lot of questions. They were the ones that always calmed us down and told us, like, ‘this is our plan. He’s going to be okay. We really think that this is going to work.’ We really just had to listen to them | Desire for more knowledge and resources regarding IEI |  |  |
| Margolis 2017 | Qualitative | Patient | "Have a conference at NIH for adolescents with CGD." | Desire for more knowledge and resources regarding IEI |  |  |
| Margolis 2017 | Qualitative | Patient | “[The doctor] pesters me and she’s persistent and I don’t want to disappoint her. I finally got myself organized. The doctors motivated me in the past few years and I started taking care of things myself.” | Desire for more knowledge and resources regarding IEI |  |  |
| Margolis 2017 | Qualitative | Patient | “A pamphlet outlining what goes into a visit starting with travel, reservations, clinic appointment.” | Desire for more knowledge and resources regarding IEI |  |  |
| Margolis 2017 | Qualitative | Patient | “Do more walk-throughs on the unit to get familiar with the unit and nurses.” | Desire for more knowledge and resources regarding IEI |  |  |
| Margolis 2017 | Qualitative | Patient | “Encourage patients to be an active participant. It’s important to know what and why.” | Desire for more knowledge and resources regarding IEI |  |  |
| Margolis 2017 | Qualitative | Patient | “Have the doctor tell parents to talk to their kids at age 15 or 16.” | Desire for more knowledge and resources regarding IEI |  |  |
| Margolis 2017 | Qualitative | Patient | “Make sure the patient is comfortable in the setting they are in. They need to (1) understand the importance of taking medicine, (2) if getting sick contact the doctor right away. (3) Understanding the disease is highly important and (4) Know it is possible to live a full healthy lifestyle if staying on top of what they need to know.” | Desire for more knowledge and resources regarding IEI |  |  |
| Margolis 2017 | Qualitative | Patient | “My parents always encouraged me to ask questions. The younger the better. My mom got scolded by the doctors for not taking a more active role but she said she wasn’t always going to be around.” | Desire for more knowledge and resources regarding IEI |  |  |
| Margolis 2017 | Qualitative | Patient | “Sit down one on one with your doctor to talk about your meds and disease.” | Desire for more knowledge and resources regarding IEI |  |  |
| Margolis 2017 | Qualitative | Patient | “Start young, like around 15 or 16, a few years before. Talk about their flights and appointments with their parents there but not doing it for them.” | Desire for more knowledge and resources regarding IEI |  |  |
| Ouimet 2023 | Qualitative | Caregiver | “But that too, the studies on this syndrome, I have books, I even ordered a book from the United States, it is until adulthood. But once it concerns adulthood, there’s nothing left.” | Desire for more knowledge and resources regarding IEI |  |  |
| Ouimet 2023 | Qualitative | Caregiver | “didn’t know that once at the adult hospital, that he [her youth] wasn’t going to have a [hospital] room for himself [ … ] We didn’t know, I don’t think even my husband knew.” | Desire for more knowledge and resources regarding IEI |  |  |
| Ouimet 2023 | Qualitative | Caregiver | Some participants did not know that the transition occurred at 18 years old and thought that “in certain cases [the pediatric] hospital kept the child until 21 years old”. As this caregiver further explained: “It’s weird, we knew 18 was the adult age, but we thought since our daughter has a mental age of seven, eight or whatever, that they would keep her for her exceptional conditions. But no.” | Desire for more knowledge and resources regarding IEI |  |  |
| Raspa 2020 | Qualitative | Caregiver | “Throughout the journey, the bone marrow transplant makes you feel so helpless. If there is no donor what do you do? You just keep waiting. I feel like a lot of education work needs to be done in that area.” | Desire for more knowledge and resources regarding IEI |  |  |
| Raspa 2020 | Quantitative | Caregiver | Caregivers generally desire additional information regarding the screening process, expectations for their child's condition and management, treatment options and their side effects, access to specialists, ways to optimize their child's wellbeing, where to seek financial assistance, risk of having another child with a PID, how to educate family and friends about their child's condition, and how to education future providers about their child's condition | Desire for more knowledge and resources regarding IEI |  |  |
| Reid 2014 | Quantitative | Patient/Caregiver | Patients and caregivers generally ranked a lack of supervision as the primary concern related to switching to home-based SCIg, followed by costs, frequent injections, lost time, having to poke themselves, and supply storage | Desire for more knowledge and resources regarding IEI |  |  |
| Smith 2021 | Qualitative | Caregiver | “I really just remember praying he would survive. But once he was through the transplant, I was expecting him not to need IVIGb and that was not the case.” | Desire for more knowledge and resources regarding IEI |  |  |
| Smith 2021 | Qualitative | Caregiver | “Our little one has normal counts of B-cells so we assumed he wouldn’t need SCIGc, now we are finding out it is because all those B-cells are his and don’t function properly because of the SCID, that he will need IgG replacement for life.” | Desire for more knowledge and resources regarding IEI |  |  |
| Wasserman 2017 | Quantitative | Patient | Some patients felt their provider explained the different routes of administration in a hurried, unclear or confusing manner, and a minority of patients felt that the routes of administration decision was made jointly between the patient and physician | Desire for more knowledge and resources regarding IEI |  |  |
| AhmadAzahari 2023 | Qualitative | Caregiver | …but it was his (child) of luck when we get to meet the right doctor who specialises in that specific field (PID)… | Preference for HCPs knowledgeable about IEI |  |  |
| AhmadAzahari 2023 | Qualitative | Caregiver | …even in the hospital if we tell them about SCID, they don’t understand, then who are we to explain to the specialist…imagine O&G specialist told me that SCID was not a genetic disease. | Preference for HCPs knowledgeable about IEI |  |  |
| AhmadAzahari 2023 | Qualitative | Caregiver | …I think the awareness with the doctors is important. | Preference for HCPs knowledgeable about IEI |  |  |
| AhmadAzahari 2023 | Qualitative | Caregiver | During my first child, I felt that it took a long time to confirm and detect the disease (SCID). But according to the doctor in the Alor Setar, Kedah area, they mentioned that now there are new specialists who are focusing on PID, specifically the disease related to PID. | Preference for HCPs knowledgeable about IEI |  |  |
| Franzese 2025 | Qualitative | Patient | “They actually sent me out a nurse who had never used the pump before. And I basically knew more about it than she did. She was actually Googling how to use the pump. And so that was quite discouraging.” | Preference for HCPs knowledgeable about IEI |  |  |
| Hansen 2004 | Quantitative | Patient | All patients percieved physicians involved with their prenatal care to have insufficient knowledge about PAD or IgRT and most patients percieved midwives to have insufficient knowledge about PAD or IgRT | Preference for HCPs knowledgeable about IEI |  |  |
| Hansen 2004 | Qualitative | Patient | I always feel a little bit silly when I have to explain about my antibody deficiency, as if it is not a “real” disease. I wish the midwives and the physicians at the prenatal clinics had known more about antibody deficiencies. | Preference for HCPs knowledgeable about IEI |  |  |
| Hansen 2004 | Qualitative | Patient | I wish the midwives had been less worried all the time and that the midwives and the physicians had listened to me and to my knowledge of my disease. | Preference for HCPs knowledgeable about IEI |  |  |
| Hansen 2004 | Qualitative | Patient | No one knew anything about the IgG therapy and when I tried to get the physicians to understand that I don’t react like others do when I get an infection and am put on “weak” antibiotics, my information was ignored. | Preference for HCPs knowledgeable about IEI |  |  |
| Hansen 2004 | Qualitative | Patient | The physician said that I should not continue with the IgG therapy now that I was pregnant. | Preference for HCPs knowledgeable about IEI |  |  |
| Hansen 2004 | Qualitative | Patient | They thought I had AIDS when they read my medical record. They were very incompetent. | Preference for HCPs knowledgeable about IEI |  |  |
| Janssen 2021 | Qualitative | Patient | The stupid thing is that there is a pulmonology guideline for recurrent airway infections. According to the protocol he had to make a referral. He didn’t know that but then at least be honest enough to say that you don’t know. | Preference for HCPs knowledgeable about IEI |  |  |
| Keestra 1988 | Quantitative | Patient | A majority of patients consider a specialist at an academic center to be the most trusted physician for their condition | Preference for HCPs knowledgeable about IEI |  |  |
| Kutsa 2022 | Qualitative | Caregiver | And then when they found out when the first transplant didn’t work, I said, let’s look at [child’s brother as a donor], you know, to see if he’s a match. And they didn’t want to do that [due to concerns about sibling’s size and age]. They said, ‘We can’t do it. Let’s do the stem cell [referring to HSCT with cord blood that was 5 out of 6 match].’ So, I had to push, I had to ask questions and keep looking. And I saw a status up on the Facebook group, and that kind of directed me and sent me to the doctors that had the experience that said, ‘yes, [sibling] could be the donor.’ There’s a lot of success with cord blood. But since my [child] had a better alternative [the sibling], and [the doctor she found via the Facebook group] said it could be done…that’s where everything went successful. | Preference for HCPs knowledgeable about IEI |  |  |
| Kutsa 2022 | Qualitative | Caregiver | When we [the child] were diagnosed, we were sent to [Hospital Name] in [City]. And they had a wonderful team there and very knowledgeable, you know, they were answering all our questions and help put our mind at ease. But it was so new to us that, well, like it is probably to most families. | Preference for HCPs knowledgeable about IEI |  |  |
| Machado 2024 | Quantitative | Patient/Caregiver | A majority of patients and caregivers cited lack of awareness of HLH, lack of specialist knowledge on the signs/symptoms of HLH, and lack of awareness of HLH by doctors as the most common causes of frustration in their diagnosis journey | Preference for HCPs knowledgeable about IEI |  |  |
| Ouimet 2023 | Qualitative | Caregiver | “[ … ] it kind of threw me for a loop when the coordinator told me … Because I said, ‘Do you have any doctors to suggest afterwards?’ She said, ‘No, now you’re falling into a pfft.’ When I told her ‘Doctors who know about velocardiofacial syndrome’ she said, ‘Good luck.” So that’s when I went ‘oh boy. Oh boy, okay. [ … ] That’s worrisome.’” | Preference for HCPs knowledgeable about IEI |  |  |
| Ouimet 2023 | Qualitative | Caregiver | “…when my daughter is sick, no matter what I say, no one understands what I’m saying, it’s like I’m speaking another language! It’s fun to be in places where people understand what you’re going through. You can go consult anywhere for cancer, but for PANDAS, you can’t go anywhere.” | Preference for HCPs knowledgeable about IEI |  |  |
| Ouimet 2023 | Qualitative | Patient | “For sure, it was like a little bit of an upheaval to know that basically I didn’t have the right diagnosis and that all the therapies that I had done before and the medication and everything, it’s kind of like I did them for nothing.” | Preference for HCPs knowledgeable about IEI |  |  |
| Ouimet 2023 | Qualitative | Patient | “I know my situation; I’ve been living with myself for 21 years. I came in with a mouth infection. They wanted to give me something because I couldn’t walk too much, I had a big arthritis crisis, they gave me fentanyl, morphine, Dilaudid. Dilaudid thins the blood. I have a blood factor that is complicated, you can’t give me a blood thinner. If I fall, I cut myself or whatever, I bleed out. So, I tell them. He says, “Well anyway, that’s not what we were ordered to do.” I said, “Fine, but I’m not taking your pill. You can write in the file that I refuse it.” So you know, you have to fight constantly, constantly. Like I tell them I’m allergic to latex. “Ah it’s not a big deal.” They put on latex gloves. It’s because I become swollen all over. You know your situation, but they’re not listening to you at all.” | Preference for HCPs knowledgeable about IEI |  |  |
| Ouimet 2023 | Qualitative | Caregiver | “The doctors really delayed prescribing medication to him because they stayed there just to observe him. They had observed him for two months without giving him anything except some kind of painkillers and that’s it. But not to cure, it’s just to control, calm, but not to cure. The disease kept developing during that time. [ … ] I think it left him with a lot of sequelae.” | Preference for HCPs knowledgeable about IEI |  |  |
| Reid 2014 | Quantitative | Patient/Caregiver | Recommendation from a clinical immunologist was related to more willingness to swap from hospital to home based IVIG | Preference for HCPs knowledgeable about IEI |  |  |
| Reid 2014 | Quantitative | Patient/Caregiver | Recommendation from a clinical immunologist was related to more willingness to swap from hospital to home based SCIg | Preference for HCPs knowledgeable about IEI |  |  |
| Reid 2014 | Quantitative | Patient/Caregiver | Recommendation from a family physician was related to more unwillingness to swap from hospital to home based IVIG | Preference for HCPs knowledgeable about IEI |  |  |
| Reid 2014 | Quantitative | Patient/Caregiver | Recommendation from a family physician was related to more unwillingness to swap from hospital to home based SCIg | Preference for HCPs knowledgeable about IEI |  |  |
| Wasserman 2017 | Quantitative | Patient | Some patients felt their provider explained the different routes of administration in a hurried, unclear or confusing manner, and a minority of patients felt that the routes of administration decision was made jointly between the patient and physician | Preference for HCPs knowledgeable about IEI |  |  |
| Wolf 2026 | Qualitative | Caregiver | “We were lucky to end up at a university center. Without that referral, we might still be waiting.” | Preference for HCPs knowledgeable about IEI |  |  |
| AhmadAzahari 2023 | Qualitative | Caregiver | During my first child, I felt that it took a long time to confirm and detect the disease (SCID). But according to the doctor in the Alor Setar, Kedah area, they mentioned that now there are new specialists who are focusing on PID, specifically the disease related to PID. | Concerns regarding delay/accuracy in diagnosis of IEI |  |  |
| AhmedMeelad 2022 | Qualitative | Caregiver | He also took medication for TB, but it did not work because it is not TB… I did not count those treatments as not helping but it's just at that point in time, the doctors had not found the real underlying cause of his problem. | Concerns regarding delay/accuracy in diagnosis of IEI |  |  |
| AhmedMeelad 2022 | Qualitative | Caregiver | It is like she has one, but the doctor did not properly diagnose her yet. | Concerns regarding delay/accuracy in diagnosis of IEI |  |  |
| Beers 2025 | Qualitative | Patient | “What I have still is just kind of like unknown, and I think that's what's the hardest part. … I've just accepted that whether it's got some acronym or not, it doesn't really matter.” | Concerns regarding delay/accuracy in diagnosis of IEI |  |  |
| Ouimet 2023 | Qualitative | Patient | “For sure, it was like a little bit of an upheaval to know that basically I didn’t have the right diagnosis and that all the therapies that I had done before and the medication and everything, it’s kind of like I did them for nothing.” | Concerns regarding delay/accuracy in diagnosis of IEI |  |  |
| Pursey 2025 | Qualitative | Caregiver | "I think I have a bit of trauma over not wanting anything else missed [after delayed diagnosis]” | Concerns regarding delay/accuracy in diagnosis of IEI |  |  |
| Pursey 2025 | Qualitative | Caregiver | "If we knew from newborn screening, we would have kept him isolated. Maybe he wouldn’t have gotten sick. Then you think what life would have been like.” | Concerns regarding delay/accuracy in diagnosis of IEI |  |  |
| Wolf 2026 | Qualitative | Caregiver | “We were lucky to end up at a university center. Without that referral, we might still be waiting.” | Concerns regarding delay/accuracy in diagnosis of IEI |  |  |
| Wolf 2026 | Qualitative | Caregiver | We were in and out of hospitals for years. Nobody thought to check the immune system. Only when our new pediatrician ran more comprehensive tests did things move forward.” | Concerns regarding delay/accuracy in diagnosis of IEI |  |  |

**Supplementary Material 10.** Thematic synthesis for review theme of “Patients and caregivers have a significant concern about the financial impact of IEI management.”

| **Study** | **Data Type** | **Perspective** | **Quote or Qualitized Narrative Summary** | **Code** | **Descriptive Theme** | **Overall Analytical Theme** |
| --- | --- | --- | --- | --- | --- | --- |
| AhmedMeelad 2022 | Qualitative | Caregiver | I would not only have a financial problem because we are only gardeners | Concern about financial burden of IEI management | Concern about financial burden of IEI management | Patients and caregivers have a significant concern about the financial impact of IEI management |
| AhmadAzahari 2023 | Qualitative | Caregiver | …I am not aware about the cost and was not informed about it either (cost for genetic testing). | Concern about financial burden of IEI management |  |  |
| AhmadAzahari 2023 | Qualitative | Caregiver | …they want to send my blood sample to see if I am a carrier, but the test needs to be sent to Australia and will cost around RM30k, so for me it is very much impossible, and I keep quiet for over 26 years. | Concern about financial burden of IEI management |  |  |
| AhmadAzahari 2023 | Qualitative | Caregiver | …we want to focus our financial for our child and if I just want to know my carrier status, it cannot solve my child condition. | Concern about financial burden of IEI management |  |  |
| AhmedMeelad 2022 | Qualitative | Caregiver | The most important thing is that our work are secured because we didn't take a lot of leave | Concern about financial burden of IEI management |  |  |
| Clark 2025 | Qualitative | Caregiver | “Financially it’s been tough. We buy her special formula and her medication, hospital parking…” | Concern about financial burden of IEI management |  |  |
| Clark 2025 | Qualitative | Caregiver | “…we probably have ended up borrowing quite a bit of money just for health, that’s a big part of our life.” | Concern about financial burden of IEI management |  |  |
| Davidson 2024 | Qualitative | Patient | Pretty much right after that, we were, like, having to jump back in to, "We got to figure this out.” … We found out that they could do, like, genetic PGD, pre-implantation genetic diagnosis, and there was no probe that had been created yet for GATA2 … There was not even, like, there wasn’t even a glimmer of hope that insurance was going to cover any of this, even though we were like, “His dad died from this. There’s family members who have died from this … [Pursuing IVF with PGD] would be preventative.” | Concern about financial burden of IEI management |  |  |
| Duff 2014 | Quantitative | Patient | Some patients reported that cost was a concern about SCIg | Concern about financial burden of IEI management |  |  |
| Eltan 2022 | Quantitative | Patient | A majority of patients reported that the treatment being financially advantageous compared to its counterparts was a reason for being satisfied with SCIg | Concern about financial burden of IEI management |  |  |
| Jones 2020b | Qualitative | Patient | "It is a pain to be fair, parking every time I come. It's not as bad now, but when I used to have the sub-cut, and I used to come to the hospital, it’s like £5 every week” | Concern about financial burden of IEI management |  |  |
| Jones 2020b | Qualitative | Patient | “At the time, I was very career-minded. I got a really, I got a really good job at the local authority at ————- Council, and I was quite high up. But then at that time, I was getting worse and worse and worse and I was having time and time off work. And obviously you can’t have, so I was forced to leave my work” | Concern about financial burden of IEI management |  |  |
| Jones 2020b | Qualitative | Patient | “I’ve had opportunities to go over to America, working over there, and I’m like I’ve got to have treatment, and travel insurance and that, they’re not going to cover the cost of this treatment. For me to pay for it, it’s probably really, from a financial standpoint- it’s just causing me a lot of problems really” | Concern about financial burden of IEI management |  |  |
| Jones 2020b | Qualitative | Patient | “It does bother me. It does because what they’ll do is they’ll sack me. That’s it, that’s the bottom line. That’s the way they are. It’s cost-cutting isn’t it. I’ve been there so long. I earn the most money of my grade amongst about 8 or 9 of us. If they can see a chance, you’re gone, and that’s what they’ll use to get rid of me. But it’s not my fault, and I’ve tried to explain this to them” | Concern about financial burden of IEI management |  |  |
| Jones 2020b | Qualitative | Patient | “So I virtually got 30 to 40 miles worth of petrol, plus £3.70 to park. So that’s what the cost” | Concern about financial burden of IEI management |  |  |
| Ouimet 2023 | Qualitative | Patient | “I had another job three years ago and they just didn’t understand. I asked for a leave of absence for the hospital, and they fired me because it was too much for them.” | Concern about financial burden of IEI management |  |  |
| Ouimet 2023 | Qualitative | Patient | “When I was young, I used to say, “I can’t wait to be 18 and an adult”, but what a shitty idea I had. […] You know, when you’re an adult, you have bills to pay, you work to pay your bills, to pay for food and then rent. I’m working right now to pay for medicine, my rent, my food. What do I have left? 25 dollars a week. It’s a shame. […] That’s basic survival. Right now, that’s the only thing I’m doing, surviving.” | Concern about financial burden of IEI management |  |  |

**Supplementary Material 11.** Thematic synthesis for review theme of “Patients and caregivers place a high value on the integration of mental health and social supports into their care.”

| **Study** | **Data Type** | **Perspective** | **Quote or Qualitized Narrative Summary** | **Code** | **Descriptive Theme** | **Overall Analytical Theme** |
| --- | --- | --- | --- | --- | --- | --- |
| Beers 2025 | Qualitative | Patient | “Diving into therapy and learning how to manage stress and all the self-care aspects for sure so I wouldn't spiral and get sick. … We need to figure out what I need to do to help myself.” | Desire for mental health, emotional, and social supports | Desire for mental health, emotional, and social supports | Patients and caregivers place a high value on the integration of mental health and social supports into their care |
| Davidson 2024 | Qualitative | Patient | It’s … a little bit anxiety-inducing because you don’t know [if you will develop symptoms] at this point. Like, at first it was like no big deal, because …my counts were all really good still. And now they’re sort of like medium to bad, but I feel fine … It’s like any year it could be the year where they’re like, “It’s go time” … So it is a weird sort of cadence to my life of like, I don’t feel like I can like, plan more than 12 months in advance. | Desire for mental health, emotional, and social supports |  |  |
| Janssen 2021 | Qualitative | Patient | Of course when being younger I really felt alone, sometimes even depressed. | Desire for mental health, emotional, and social supports |  |  |
| Jones 2020b | Qualitative | Patient | “I used to go a day a week into hospital and then eventually I was on home therapy and that were fantastic. I do miss, miss them though. I said to [name deleted] because I used to go in one day a week, and you get so looked after, and because you’re chatting to other patients and other people, and you’re watching all the people, and when you go on home therapy you do lose that” | Desire for mental health, emotional, and social supports |  |  |
| Margolis 2017 | Qualitative | Patient | “Have a support system with other patients. Talking to other patients about transition would help. I would be willing to talk to others.” | Desire for mental health, emotional, and social supports |  |  |
| Margolis 2017 | Qualitative | Patient | “I think it’s best to have similar people your age ease you into the situation. Talking to someone personal would be really helpful. Someone to connect with on your level that knows what you’re going through. I’ve found it useful to talk to other patients with CGD.” | Desire for mental health, emotional, and social supports |  |  |
| Pursey 2025 | Qualitative | Caregiver | “I think preparing families, it’s a long road. You don’t just get to have transplant and go home.” | Desire for mental health, emotional, and social supports |  |  |
| Pursey 2025 | Qualitative | Caregiver | “I think that’s the first time I really panicked, because I felt like nobody was listening” | Desire for mental health, emotional, and social supports |  |  |
| Raspa 2020 | Qualitative | Caregiver | “His type of SCID is very rare so we didn’t know what was going to be the right course of action for him. There was nothing helpful to me. Maybe if parents could be provided brochures with different families’ testimonies and stories. I think that would be very helpful for parents going through it now. I didn’t know there were so many varieties of SCID and so many treatments for each category. Some can get the newer ones now. I didn’t have any of that information.” | Desire for mental health, emotional, and social supports |  |  |
| Raspa 2020 | Quantitative | Caregiver | Caregivers generally desire the opportunity to talk to other families and support groups knowledgeable in their child's condition, options and supports to manage the changes in relationship with their family and partner, and access to professional counselling and other emotional management options | Desire for mental health, emotional, and social supports |  |  |

**Supplementary Material 12.** GRADE-CERQual domain rating justifications.

| **Review Finding** | **Methodology** | **Coherence** | **Adequacy** | **Relevance** | **Overall Confidence in Evidence** |
| --- | --- | --- | --- | --- | --- |
| Patients and caregivers are more willing to engage with and accept treatment if counseled by HCPs knowledgeable in IEI | **Moderate concerns**  Many studies with few methodological concerns. Researcher and participant relationship sometimes not reported. Some studies' data analysis appeared to be insufficiently rigorous. Attrition rates and pre-piloting of surveys rarely reported. | **Minor concerns**  Findings are consistent across all studies. Studies address this theme in slightly different ways; some state that patients valued strong relationships and counselling with their care team, and others reported that patients dislike a lack of information from their care team. | **No or very minor concerns**  Theme is adequately addressed with supporting data from many studies. | **No or very minor concerns**  Perspectives of patients in several countries represented. Studies include general IEI patients and specific IEI diseases. | Moderate confidence |
| Patients and caregivers place a high value on IEI treatments that minimize needle pricks and adverse effects | **Moderate concerns**  Many studies with few methodological concerns. Researcher and participant relationship sometimes not reported. Some studies' data analysis appeared to be insufficiently rigorous. Attrition rates and pre-piloting of surveys rarely reported. | **No or very minor concerns**  Findings are consistent and unambiguous across all studies. | **No or very minor concerns**  Theme is adequately addressed with supporting data from many studies. | **No or very minor concerns**  Perspectives of patients in several countries represented. Studies include general IEI patients and specific IEI diseases. | Moderate confidence |
| Patients and caregivers place a high value on IEI treatments that maximize independence, efficiency, and flexibility | **Moderate concerns**  Many studies with few methodological concerns. Researcher and participant relationship sometimes not reported. Some studies' data analysis appeared to be insufficiently rigorous. Attrition rates and pre-piloting of surveys rarely reported. | **Minor concerns**  A few studies cited that patients and caregivers prefer hospital-based IgRT over home-based IgRT due to greater access to HCPs | **No or very minor concerns**  Theme is adequately addressed with supporting data from many studies. | **No or very minor concerns**  Perspectives of patients in several countries represented. Studies include general IEI patients and specific IEI diseases. | Moderate confidence |
| Patients and caregivers place a high value on effective transitions from pediatric to adult care services | **Moderate concerns**  Many studies with few methodological concerns. Researcher and participant relationship sometimes not reported. Some studies' data analysis appeared to be insufficiently rigorous. Attrition rates and pre-piloting of surveys rarely reported. | **No or very minor concerns**  Findings are consistent and unambiguous across all studies. | **No or very minor concerns**  Theme is adequately addressed with supporting data from many studies. | **No or very minor concerns**  Perspectives of patients in several countries represented. Studies include general IEI patients and specific IEI diseases. | Moderate confidence |
| Patients and caregivers have a significant concern about the financial impact of IEI management | **Moderate concerns**  Many studies with few methodological concerns. Researcher and participant relationship sometimes not reported. | **No or very minor concerns**  Findings are consistent and unambiguous across all studies. | **Moderate concerns**  Only a few studies address this theme, and there is a limited amount of supporting data. | **No or very minor concerns**  Perspectives of patients in several countries represented. Studies include general IEI patients and specific IEI diseases. | Low confidence |
| Patients and caregivers place a high value on the integration of mental health and social supports into their care | **Moderate concerns**  Many studies with few methodological concerns. Attrition rates and pre-piloting of surveys not reported. | **Minor concerns**  A few studies cited that patients and caregivers found the financial impact of IEI to be manageable | **Moderate concerns**  Only a few studies address this theme, and there is a limited amount of supporting data. | **No or very minor concerns**  Perspectives of patients in several countries represented. Studies include general IEI patients and specific IEI diseases. | Low confidence |
